# Hierarchical Mission Planning with a GA-Optimizer for Unmanned High Altitude Pseudo-Satellites

**DOI:** 10.3390/s21051630

**Published:** 2021-02-26

**Authors:** Jane Jean Kiam, Eva Besada-Portas, Axel Schulte

**Affiliations:** 1 Institute of Flight Systems, Bundeswehr University Munich, 85579 Neubiberg, Germany; axel.schulte@unibw.de; 2 Department of Computer Architecture and Automation, Universidad Complutense de Madrid, 28040 Madrid, Spain; ebesada@ucm.es

**Keywords:** HAPS, UAV, monitoring, constrained multiple objective optimization, temporal hierarchical task planning

## Abstract

Unmanned Aerial Vehicles (UAVs) are gaining preference for mapping and monitoring ground activities, partially due to the cost efficiency and availability of lightweight high-resolution imaging sensors. Recent advances in solar-powered High Altitude Pseudo-Satellites (HAPSs) widen the future use of multiple UAVs of this sort for long-endurance remote sensing, from the lower stratosphere of vast ground areas. However, to increase mission success and safety, the effect of the wind on the platform dynamics and of the cloud coverage on the quality of the images must be considered during mission planning. For this reason, this article presents a new planner that, considering the weather conditions, determines the temporal hierarchical decomposition of the tasks of several HAPSs. This planner is supported by a Multiple Objective Evolutionary Algorithm (MOEA) that determines the best Pareto front of feasible high-level plans according to different objectives carefully defined to consider the uncertainties imposed by the time-varying conditions of the environment. Meanwhile, the feasibility of the plans is assured by integrating constraints handling techniques in the MOEA. Leveraging historical weather data and realistic mission settings, we analyze the performance of the planner for different scenarios and conclude that it is capable of determining overall good solutions under different conditions.

## 1. Introduction

Regular monitoring of land development (e.g., agricultural activities, big construction sites, essential infrastructure, wildforest, etc.) can be done using either satellites or airplanes. Recently, Unmanned Aerial Vehicles (UAVs) are preferred for a more cost-efficient and flexible deployment. However, UAVs flying at low altitude may not always be a solution, as their missions depend on the possibility of obtaining a permit-to-fly, on weather conditions that can be quite challenging at low altitude, and on the required flight range. In the case of fixed-wing UAVs, the takeoff and landing can also be troublesome for regular deployments or may not even be an option from surroundings with unfavorable topologies.

Solar-powered unmanned High Altitude Pseudo-Satellites (HAPSs) are considered a viable alternative to overcome the challenges arising from using satellites with a fixed orbit, manned airplanes, or UAVs for regular monitoring. As [1] explains, HAPSs are a type of light-weight High Altitude Long Endurance (HALE) aerial platforms that fly at low speed (in order to be energy efficient), in the lower stratosphere (where the airspace is quite calm and little congested), with extremely long endurance (e.g., [2] reports a continuous HAPSs flight of almost 26 days). Moreover, although still at its infancy, the development of HAPSs is promising and is expected to provide multiple benefits. However, given their light-weight build, operating HAPSs can also be challenging. Table 1 summarizes some of the general benefits (+) and challenges (−) of these platforms, according to the relevant characteristics of the HAPSs that contribute to each of them.

HAPSs operations contain the typical space flight phases, such as planning, processing, departure and flight operations, return and landing, refurbishment, and turnaround [3]. However, these phases present some peculiarities, due to the HAPSs characteristics. For instance, and according to the analysis presented in [4] on the trajectories obtained from a test flight conducted using the Kelleher platform in Arizona in 2018, this HAPS takes around a day to ascend/descend to/from its operating altitude by flying within a safe vertical corridor allocated for takeoff and landing. Subsequently, the platform stays as long as possible in the air and at the operating altitude in the lower stratosphere.

Given these continuous and extremely long operations, increasing HAPSs autonomy is essential. Besides, it is also useful from a safety and pragmatic point of view, as well as to reduce manpower and human error. Finally, and according to [5], the deployment of HALE platforms can be “cost-efficient”, since by increasing autonomy and decreasing piloting, operation cost and be further reduced (without compromising safety and efficiency).

Hence, automated mission planning is convenient for the deployment of HAPSs that have to perform monitoring missions. However, although the airspace at this flight level is often relatively calm with mild winds, some rare weather conditions can pose serious safety-critical risks to the HAPSs. Moreover, since these platforms have limited maneuverability, reactive avoidance of risk zones may not always be possible. Therefore, weather risks must be addressed already in the mission planner on the Ground Control Station (GCS) to minimize the need of an onboard replanning or emergency landing. In particular, the following weather conditions must be considered:

- Cumulonimbus clouds: Although clouds are rare in the stratosphere where HAPSs operate, the anvil of Cumulonimbus clouds can reach high altitudes and is extremely dangerous. Hence, it must be avoided with substantial distance (∼37 km laterally and 1.5 km vertically) to prevent structural impairment to the platforms [6].
- Turbulences and Precipitation: These weather phenomena can be caused by strong winds and wind shear [7]. Although rare and harmless to bigger aircraft (e.g., airliners), turbulences and precipitations can cause extreme difficulties to HAPSs navigation and damage their structures.
- Wind field: Given the low airspeed of the HAPS, even mild wind (with wind speed up to 5 m/s) must be considered in planning for wind drift correction.

Besides, and although HAPSs airspace is little congested (since the airliners fly below), High-level Flight Rules (HFR) also apply to unmanned flights above Flight Level (FL) 600 [8]. This implies that the airspace regulations must also be considered in the mission planner (in order to avoid collisions with other stratospheric aircrafts), that it is recommendable to systematically organize and dynamically allocate the airspace [9], and that the flight plans must be communicated to the authorities before the execution of each mission. However, since HAPSs are long-endurance platforms intended to remain airborne, planning must also be performed during flight, before each monitoring mission starts.

Taking into account the previous considerations, this work focuses on increasing the autonomy and efficiency in mission planning that takes place on the GCS during flight operations but before the execution of the mission-related tasks. Our main goal is to optimize the mission success rate of monitoring the requested sites (i.e., to improve the chances of providing images of the sites with sufficient coverage and at the requested time windows), while reducing the risk of replanning by considering, at the planning phase, the predicted time-varying environment and the platform constraints. Furthermore, we assume the presence of one or several human operators in the mission-planning loop. Although their decision-making process is not considered in this work, our mission planner is developed to be part of a decision-support system that is responsible for automatically generating a group of feasible optimal plans and for presenting them as “suggestions” to the operators, who have to perform the selection of the final plan.

The work presented in this paper is closely related with the approach described in [10], which presented the preliminary version of our planner. The current version is improved, by (1) adopting a Multi-Objective Evolutionary Algorithm (MOEA) for constrained problems to optimize the mission plans and (2) by considering the uncertainty associated to the wind variability in the constraints. Besides, this paper presents new scenarios and analyzes the results of the new planner over a wider set of circumstances. Finally, it is worth noting that the relationships of other works with ours will be discussed later in Section 6, after the readers are acquainted with the main characteristics of our planner described through Section 3 and Section 4.

The organization of this work is the following. Section 2 presents the problem at an abstract level and describes its main elements. Section 3 provides a more formal description of the problem, including the objective functions as well as the different components that conform with the constraint criteria. Subsequently, the implementation of the MOEA that supports the optimization process of the planner is described in Section 4, providing details on the encoding of the plan, on the hierarchical task decomposition process and on the particularities of our MOEA. Finally, results are illustrated and analyzed in Section 5, while a discussion on related work is provided in Section 6, followed by the conclusion and future work drawn in Section 7.

## 2. Problem Description

This work focuses on the task planning for multiple HAPSs, equipped with electro-optical (EO) mission cameras and contracted to monitor repeatedly areas on the ground at specific time windows.

This section presents the main elements of the problem, describing the monitoring scenario that will be considered in this paper, introducing how the mission plan is defined, characterizing the payload of the HAPSs, reporting the mission requirements and constraints, and finally, explaining how the weather conditions to be considered at the planning phase are extracted.

### 2.1. Monitoring Scenarios

**Figure 1:**
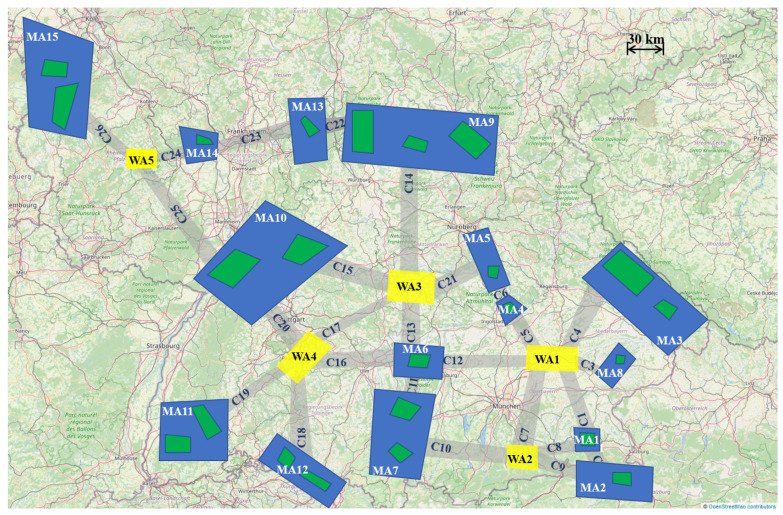
Mission scenario (plotted on *©* OpenStreetMap) for monitoring multiple Locations Of Interest (LOI#) on the ground. The operation airspace is organized using dynamically allocated mission elements of Corridors (C#), Waiting Areas (WA#), and Mission Areas (MA#) that encompass LOIs.

The operation is assumed to take place in an organized airspace consisting of different types of dynamically allocated operation areas, in order to reduce congestion in the lower stratosphere. In particular, and as shown in Figure 1, the HAPSs will be able to operate in Mission Areas (MAs, represented in blue), Corridors (Cs, in gray), and Waiting Areas (WAs, in yellow).

Besides, the Locations of Interest (LOIs, in green) are the projection of the ground areas to be monitored within the time windows and at the frequency requested by the clients. LOIs of the same client with the same set of mission requirements are grouped in a MA, which defines the airspace (at the operating altitude for the HAPSs), allocated to allow a HAPS to monitor the encompassed group of LOIs. Additionally, the WAs are airspace made available for the HAPSs to loiter freely (for example upon sunset) or to exploit as a “corridor” to reach another connected MA. HAPSs are allowed to move between MAs only through the designated Cs or through WAs. This also implies that MAs are not to be used as “corridors”, i.e., a HAPS entering a MA has to monitor its corresponding LOIs before departing through a connected corridor.

Appendix A includes further details of the HAPSs considered in this work and of the mission scenario represented in Figure 1. In particular, the numerical information on the model of the HAPSs is adapted from [11] and summarized in Table A1, while the dimensions of the mission elements are presented in Table A2.

### 2.2. Hierarchical Task Plan

Execution of tasks for multiple HAPSs can be structured conveniently in a hierarchical manner, since the order of task execution depends substantially on the organisation of the airspace and on airspace-related constraints and requirements, which can be expressed at different levels of spatial resolution. In particular, we consider the following levels, ordered from the highest to the lowest level, according to the spatial resolution:

1. MA level, where the plans are the sequences of mission areas (MA#) and waiting areas (WA#) that each HAPS operates.
2. LOI level, where the plans are sequences of tasks to be performed in the mission elements expressed at one higher abstraction level (i.e., MA# and WA#). Examples of these tasks are flying through a WA (fly${}_{\mathrm{WA}\#}$) and monitoring a LOI in a given MA (monitor${}_{\mathrm{LOI}\#\in \mathrm{MA}\#}$).
3. Waypoint (WP) level, where the plans consist of either executing a scan pattern (scan) over a LOI or flying to sequences of waypoint, which are: fly to the closest entrance of a given corridor (to${}_{C\#}$), cross and fly to the end location of the given corridor (cross${}_{C\#}$), and fly to the closest vertex of the LOI that has to be monitored (NPL).

**Figure 2:**
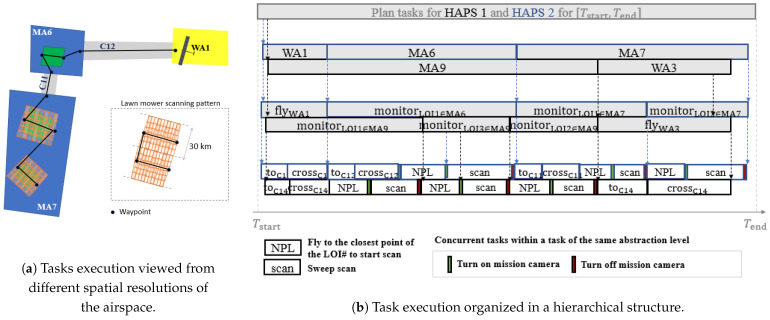
Hierarchical task execution for HAPS.

Figure 2 illustrates the representation of a hierarchical task plan considered in this work. In particular, on the left side, Figure 2a shows, over a portion of the mission scenario, the execution of the task plan for a HAPS that, after the first task in WA1, continues monitoring first the unique LOI in MA6 and afterwards the two LOIs in MA7. This figure also shows the waypoints followed by the HAPS to move within MA1 and MA7. On the right side, Figure 2b shows the hierarchical structure of the task plan of the HAPS represented in Figure 2a and of a second HAPS (not depicted in Figure 2a). That is, it shows how the plans that govern the two HAPS to monitor the LOIs within the horizon [$T_{\mathrm{start}},T_{\mathrm{end}}$] are initially decomposed into the tasks expressed at the MA-level (represented at the two top timelines, one for each HAPS), followed by the tasks expressed at the LOI-level (represented at the two intermediate timelines) and finally by the actions presented at the WP-level (shown at the two bottom-most timelines). At the lowest level, vertical color bars without text annotation represent instantaneous tasks, for example, to turn on or off the mission camera.

### 2.3. Mission Payload

The HAPSs are equipped with light-weight electro-optical mission cameras. The example camera considered in this work is inspired by the one described by Delauré et al. in [12], specially designed for unmanned HALE platforms. In particular, it is a light-weight (∼2.6 kg) and energy-efficient (<50 W) camera with two custom CMOS image sensors and with resistance to low pressure (down to 60 mbar) and to a wide range of temperature (from −70 °C to 60 °C). Its pixel counts for the width $w_{I}$ and height $h_{I}$ of the image are 1200 px × 10,000 px. With a ground sampling distance of 30 cm, an image taken from an altitude of 18 km at Nadir position records an area of 360 m × 3000 m of the ground.

With this mission camera and a gimbal that performs a cross-track sweep scan within 10 s from −45${}^{\circ }$ to 45${}^{\circ }$, a HAPS can record images covering a total width of more than 30 km, while advancing forward. Figure 2a illustrates the scanned footprint, which is a superposition of images taken during the scan. Even in the presence of a tailwind of 5 m/s, the HAPS, flying at the airspeed of 30 m/s (that is considered in Table A1) will not advance more than 360 m within a cross-track sweep, assuring some overlapping of the images between two periodic sweeps. Therefore, and as Figure 2a shows, we adopt a lawn mower scan pattern to monitor each LOI, with the distance between two consecutive tracks set at 30 km.

Finally, the cloud layers between the HAPS and the ground must be considered during the monitoring scans, as they reduce the coverage of the area recorded with the EO mission camera. For this reason, if the stitched image of any one of the LOIs of a MA has a coverage of the ground lower than requested, the monitoring of that MA will not be rewarded by the client.

### 2.4. Mission Requirements

The HAPS team is rewarded by the contracting client if the ensemble of all the monitoring tasks performed on the LOIs within a MA is considered “successful”. Therefore, we consider this ensemble of tasks a “mission” unit, which is rewarded according to the amount agreed upon by each client.

In particular, monitoring a mission unit is successful if the following mission requirements (MRs) are fulfilled:

- **MR1**: The recorded image of each LOI has a coverage of the LOI that is bigger than the minimum required coverage for its corresponding MA.
- **MR2**: The captured images of each LOI are within the time windows requested by the client for each MA.
- **MR3**: The time-lapse between two consecutive successful visits to the MA is larger than the imposed minimum inter-visit time-lapse for the MA.
- **MR4**: The MA has not been visited more frequently per day than required by the client.

The coverage percentage and reward obtained for monitoring successfully each MA of the scenario presented in Figure 1 is presented in Table A3 of Appendix A. Besides, the rewarding time windows for each MA are directly depicted together with the mission plans obtained by our planner, which are presented in Figures 10–14 of Section 5, since they are required to observe if the MA can be successfully or unsuccessfully monitored. Finally, in the scenarios analyzed in this paper, the time-lapse between the start times of two consecutive successful visits is set to one hour and each MA must not be visited more than three times a day.

### 2.5. Mission Constraints

While mission requirements decide if a mission is successful, mission constraints (MCs) dictate the “feasibility” of a plan and are defined in the interest of operational safety by enforcing airspace regulation and measures for risk avoidance.

In particular, a plan is infeasible (i.e., it cannot be executed) if any of the following constraints is violated:

- **MC1**: any mission element that the HAPS is operating in has a wind field with a wind magnitude smaller than 5 m/s.
- **MC2**: the MA or WA that the HAPS is operating in (e.g., a MA, a WA, or a C) has an obstacle occlusion (related with zones of adverse weather) smaller than 30%.
- **MC3**: Only one HAPS can operate in a MA (i.e., the simultaneous coexisting of HAPSs in a MA is forbidden).
- **MC4**: Consecutive MAs or WAs have to be connected according to the mission scenario.
- **MC5**: LOIs are monitored exactly once at each visit to the MA.
- **MC6**: A MA cannot be used as a corridor, i.e., HAPS cannot pass the MA without monitoring all its encompassed LOIs.

### 2.6. Weather Conditions

Weather conditions also affect the HAPSs and can make a given mission plan unsuccessful and/or infeasible. To take them into account, high-resolution global weather forecast based on numerical weather prediction models can be used, because this approach is beneficial compared to wide area weather forecast to foresee risk zones and to consider wind effects.

In particular, for this study we use the COSMO-D2 (COnsortium for Small-scale MOdeling) numerical weather data from the German National Meteorological Service (Deutscher Wetterdienst, DWD), which are updated every couple of hours to provide information on the cloud coverage and on the wind vector field with a horizontal resolution of 2.2 km and a temporal resolution of one hour [13].

In order to argue for availability of weather data that fit the underlying framework, we also list here a set of alternative meteorological services that can be used in the mission planner described in this paper, which also provide numerical global weather data such as the Global Forecast System (GFS, Ref. [14]) and the European Center for Medium-Range Weather Forecast (ECMWF, Ref. [15]).

## 3. Formal Problem Statement

This section defines the problem formally, detailing the variables used to mathematically define a hierarchical plan, as well as the objective and the constraint functions used to evaluate them.

**Figure 3:**
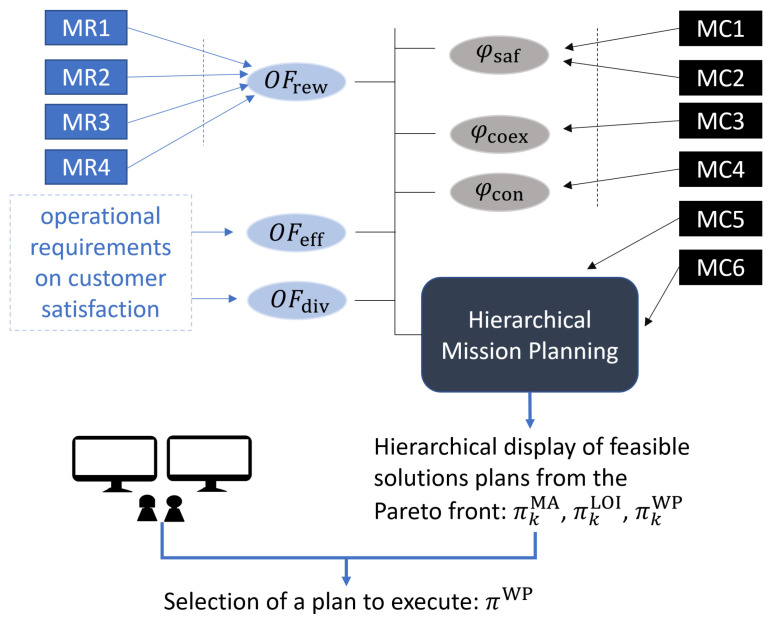
Relationships among the mission requirements (MRs) and mission constraints (MCs) described in Section 2 and the objective functions (OF) and constraints (φ) presented in Section 3.

To help the reader understand the relationship of the elements presented in this section and the previous sections, Figure 3 shows how the MRs and MCs described in Section 2.4 and Section 2.5 are mapped into the three Objective Functions ($OF_{\mathrm{rew}}$, $OF_{\mathrm{eff}}$, $OF_{\mathrm{div}}$) and the three constraint criteria ($\varphi_{\mathrm{saf}}$, $\varphi_{\mathrm{coex}}$, $\varphi_{\mathrm{con}}$) that are formally stated in this section. Furthermore, Figure 3 also illustrates the role of the operators as human decision makers, i.e., how they select a plan ($\pi_{k}^{\mathrm{MA}}$, $\pi_{k}^{\mathrm{LOI}}$, $\pi_{k}^{\mathrm{WP}}$) among the feasible solution plans that form part of the first Pareto front determined by the planner that will be presented in Section 4.

### 3.1. Formal Definition of the Hierarchical Plan

The goal of the planning problem is to find a hierarchically structured plan such as the one depicted in Figure 2b that entails the sequence of tasks to be performed by each HAPS, as well as their expected initial time instant and duration.

Formally, the solution is a set of sequences of time-stamped tasks for each hierarchical level and HAPS. More in detail:

- At the MA level, the plan can be represented as the ordered list of tasks $\pi_{h}^{\mathrm{MA}}$ displayed in Equation (1), where $o_{h,i}^{\mathrm{MA}}$ is the *i*-th mission task (i.e., a MA# or WA# of the mission scenario) on the list that will be performed by HAPS *h*, and $t_{h,i}^{\mathrm{MA},\mathrm{start}}$ and $\delta_{h,i}^{\mathrm{MA}}$ are the start time and the duration of the i−th task of HAPS *h*.
  (1) $$\pi_{h}^{\mathrm{MA}}=<o_{h,1}^{\mathrm{MA}}\left(t_{h,1}^{\mathrm{MA},\mathrm{start}},\delta_{h,1}^{\mathrm{MA}}\right),o_{h,2}^{\mathrm{MA}}\left(t_{h,2}^{\mathrm{MA},\mathrm{start}},\delta_{h,2}^{\mathrm{MA}}\right),o_{h,3}^{\mathrm{MA}}\left(t_{h,3}^{\mathrm{MA},\mathrm{start}},\delta_{h,3}^{\mathrm{MA}}\right),\cdots >$$
  Under this formulation, the high level mission plan of the first HAPS displayed in Figure 2b will be represented as $\pi_{1}^{\mathrm{MA}}$ =< WA1($t_{1,1}^{\mathrm{MA},\mathrm{start}}$, $\delta_{1,1}^{\mathrm{MA}}$), MA6($t_{1,2}^{\mathrm{MA},\mathrm{start}}$, $\delta_{1,2}^{\mathrm{MA}}$), MA7($t_{1,3}^{\mathrm{MA},\mathrm{start}}$, $\delta_{1,3}^{\mathrm{MA}}$)>.
- At the LOI-level, the plan $\pi_{h}^{\mathrm{LOI}}$ can be represented with Equation (2), where $o_{h,i}^{\mathrm{LOI}}$ is the *i*-th mission task (i.e., fly${}_{\mathrm{WA}\#}$ or monitor${}_{\mathrm{LOI}\#\in \mathrm{MA}\#}$) that will be performed by HAPS *h*, and $t_{h,i}^{\mathrm{LOI},\mathrm{start}}$ and $\delta_{h,i}^{\mathrm{LOI}}$ are the start time and duration of the i−th task of HAPS *h*.
  (2) $$\pi_{h}^{\mathrm{LOI}}=<o_{h,1}^{\mathrm{LOI}}\left(t_{h,1}^{\mathrm{LOI},\mathrm{start}},\delta_{h,1}^{\mathrm{LOI}}\right),o_{h,2}^{\mathrm{LOI}}\left(t_{h,2}^{\mathrm{LOI},\mathrm{start}},\delta_{h,2}^{\mathrm{LOI}}\right),o_{h,3}^{\mathrm{LOI}}\left(t_{h,3}^{\mathrm{LOI},\mathrm{start}},\delta_{h,3}^{\mathrm{LOI}}\right),\cdots >$$
  Under this formulation, the middle level mission plan of the first HAPS displayed in Figure 2b will be represented as $\pi_{1}^{\mathrm{LOI}}=$<${\mathrm{fly}}_{\mathrm{WA}1}$($t_{1,1}^{\mathrm{LOI},\mathrm{start}}$, $\delta_{1,1}^{\mathrm{LOI}}$), ${\mathrm{monitor}}_{\mathrm{LOI}1\in \mathrm{MA}6}$ ($t_{1,2}^{\mathrm{LOI},\mathrm{start}}$, $\delta_{1,2}^{\mathrm{LOI}}$), ${\mathrm{monitor}}_{\mathrm{LOI}1\in \mathrm{MA}7}$($t_{1,3}^{\mathrm{LOI},\mathrm{start}}$, $\delta_{1,3}^{\mathrm{LOI}}$), ${\mathrm{monitor}}_{\mathrm{LOI}2\in \mathrm{MA}7}$($t_{1,4}^{\mathrm{LOI},\mathrm{start}}$, $\delta_{1,4}^{\mathrm{LOI}}$)>. Moreover, we can relate the time variables of the MA and LOI level (e.g., $t_{1,1}^{\mathrm{LOI},\mathrm{start}}=t_{1,1}^{\mathrm{MA},\mathrm{start}}$, $t_{1,2}^{\mathrm{LOI},\mathrm{start}}=t_{1,2}^{\mathrm{MA},\mathrm{start}}$, $t_{1,3}^{\mathrm{LOI},\mathrm{start}}=t_{1,3}^{\mathrm{MA},\mathrm{start}}$, or $t_{1,4}^{\mathrm{LOI},\mathrm{start}}=t_{1,3}^{\mathrm{MA},\mathrm{start}}+\delta_{1,3}^{\mathrm{LOI}}$) to signify the decomposition of the higher level task into lower-level tasks.
- A similar representation, where $o_{h,i}^{\mathrm{WP}}$ are the actions that can be performed at the lower mission level, and $t_{h,i}^{\mathrm{WP},\mathrm{start}}$ and $\delta_{h,i}^{\mathrm{WP}}$ are its corresponding start time and duration, applies to the WP-level.
- Finally, we extend the previous notations as follows:
  – $\pi^{\mathrm{MA}}$, $\pi^{\mathrm{LOI}}$ and $\pi^{\mathrm{WP}}$ represent the plans of the set of *H* HAPSs (i.e., $\pi^{*}=<\pi_{1}^{*},\pi_{2}^{*},\cdots ,\pi_{H}^{*}>$, where * stands either for MA, LOI, or WP).
  – ${\tilde{\pi }}_{h,i:j}^{\mathrm{MA}}$, ${\tilde{\pi }}_{h,i:j}^{\mathrm{LOI}}$ and ${\tilde{\pi }}_{h,i:j}^{\mathrm{WP}}$ represent the partial plans between the *i*-th and *j*-th task (i.e., ${\tilde{\pi }}_{h,i:j}^{*}=<o_{h,i}^{*}\left(t_{h,i}^{*,\mathrm{start}},\delta_{h,i}^{*}\right),o_{h,i+1}^{*}\left(t_{h,i+1}^{*,\mathrm{start}},\delta_{h,i+1}^{*}\right),\ldots ,o_{h,j}^{*}\left(t_{h,j}^{*,\mathrm{start}},\delta_{h,j}^{*}\right)>$, where * stands either for MA, LOI, or WP.

At this point, it is necessary to highlight that the time-dependent variables ($t_{h,i}^{\mathrm{MA},\mathrm{start}},\delta_{h,i}^{\mathrm{MA}},$$t_{h,i}^{\mathrm{LOI},\mathrm{start}},\delta_{h,i}^{\mathrm{LOI}},t_{h,i}^{\mathrm{WP},\mathrm{start}},\delta_{h,i}^{\mathrm{WP}}$) are probabilistic in our problem, except for the start time of the mission $t_{0}=t_{h,1}^{\mathrm{MA},\mathrm{start}}=t_{h,1}^{\mathrm{LOI},\mathrm{start}}=t_{h,1}^{\mathrm{WP},\mathrm{start}}$. The underlying reason of the probabilistic nature of these variables is that, at the lowest spatial resolution, the duration of each task can only be estimated, since neither the trajectory of the HAPS nor the exact wind vector are computed or considered yet.

For this reason, we model the duration $\delta_{h,i}^{\mathrm{WP}}$ of a task at the WP level as a random variable *uniformly* distributed over:

(3) $$\left[\delta_{h,i}^{\mathrm{WP},\min},\delta_{h,i}^{\mathrm{WP},\max}\right]=[l\left(o_{h,i}^{\mathrm{WP}}\right)/\left(\right|v_{a}\left|+\max(\right|v_{w}|)),l\left(o_{h,i}^{\mathrm{WP}}\right)/\left(\right|v_{a}\left|-\max(\right|v_{w}|)],$$

where $l(o_{h,i}^{\mathrm{WP}})$ is the total linear distance to travel between the waypoints associated to the task $o_{h,i}^{\mathrm{WP}}$, $|v_{a}|$ is the cruising airspeed of the HAPS (see Table A1), and $\max(|v_{w}|)$ is the maximum wind magnitude (which is assumed to be 5 m/s in this study to ensure that MC1 is not violated). Hence, when we consider $|v_{a}\left|+\max(\right|v_{w}\left|\right)$ we assume that the HAPS is flying with tailwind, while by considering $|v_{a}\left|-\max(\right|v_{w}\left|\right)$, we assume that the HAPS is flying with headwind. Note that since $l(o_{h,i}^{\mathrm{WP}})$ can only be estimated upon the decomposition down to the WP level, our hierarchical planning approach searches for a plan by adopting a downward-forward decomposition approach, which will be explained in a later section in Algorithm 1.

Assuming that lingering between the tasks at any level is forbidden, task $o_{h,i}^{\mathrm{WP}}$ terminates at $t_{h,i}^{\mathrm{WP},\mathrm{end}}=t_{0}+\Sigma_{j=1}^{i}\delta_{h,j}^{\mathrm{WP}}$, where $t_{0}$ is the deterministic start time of the plan, while $\Sigma_{j=1}^{i}\delta_{h,j}^{\mathrm{WP}}$ follows the distribution of the sum of *i* nonidentically distributed uniform random variables $\delta_{h,j}^{\mathrm{WP}}$. Therefore, the probability density function $f(t_{h,i}^{\mathrm{WP},\mathrm{end}})$ of completing the i−th WP-level task of HAPS *h* at a given time can be calculated with the following expression, as derived in [16]:

(4) $$f\left(t_{h,i}^{\mathrm{WP},\mathrm{end}}\right)=f\left(t_{h,i}^{\mathrm{WP},\mathrm{end}}-t_{0}\right)=f\left(\sum_{j=1}^{i}\delta_{h,j}^{\mathrm{WP}}\right)=\frac{\sum_{{\vec{\epsilon }}^{k}\in V^{i}}\;\;{\left(g\left({\vec{\epsilon }}^{k},\delta_{h,1:i}^{\mathrm{WP}}\right)\right)}^{i-1}\;\cdot \;\mathrm{sign}\left(g\left({\vec{\epsilon }}^{k},\delta_{h,1:i}^{\mathrm{WP}}\right)\right)\prod_{j=1}^{i}\epsilon_{j}}{\left(i-1\right)\;!\;2^{i+1}\prod_{j=1}^{i}u_{\delta_{h,j}^{\mathrm{WP}}}}\;$$

where $V^{i}$ comprises the set with all $2^{i}$ vectors of signs ${\vec{\epsilon }}^{k}=\left(\epsilon_{1}^{k},\cdots ,\epsilon_{i}^{k}\right)\in {\left\{-1,1\right\}}^{i}$, $u_{\delta_{h,i}^{\mathrm{WP}}}=\left(\delta_{h,i}^{\mathrm{WP},\max}-\delta_{h,i}^{\mathrm{WP},\min}\right)/2$, i! is the factorial of *i*, and $g\left({\vec{\epsilon }}^{k},\delta_{h,1:i}^{\mathrm{WP}}\right)=\sum_{j=1}^{i}\delta_{h,j}^{\mathrm{WP}}+\sum_{j=1}^{i}\left(\epsilon_{j}u_{\delta_{h,j}^{\mathrm{WP}}}-m_{\delta_{h,j}^{\mathrm{WP}}}\right)$, with $m_{\delta_{h,i}^{\mathrm{WP}}}$ being the median value of [$\delta_{h,i}^{\mathrm{WP},\min},\delta_{h,i}^{\mathrm{WP},\max}$].

The distribution of the higher levels (LOI and MA) time-dependent random variables can be modelled, given the hierarchical decomposition of the plan and the lack of lingering between tasks, by considering the distribution of the lowest level (WP-level) time-dependent random variables, i.e.,

(5) $$f\left(t_{h,i}^{\mathrm{MA},\mathrm{end}}\right)=f\left(t_{h,j}^{\mathrm{LOI},\mathrm{end}}\right),$$

(6) $$f\left(t_{h,j}^{\mathrm{LOI},\mathrm{end}}\right)=f\left(t_{h,k}^{\mathrm{WP},\mathrm{end}}\right),$$

where $t_{h,i}^{\mathrm{MA},\mathrm{end}}$ is the end time of the i−th task of the MA level, $t_{h,j}^{\mathrm{LOI},\mathrm{end}}$ is the ending time of the j−th task of the LOI level that terminates when the i−th task of the MA level ends, and $t_{h,k}^{\mathrm{WP},\mathrm{end}}$ is the ending time of the k−th task of the WP level that terminates when the i−th task of the MA level and the j−th task of the LOI level end. In other words, the distributions of the higher levels are associated to some of the distributions of the lower ones. Finally, it is worth noting that the estimated end time $t_{h,i}^{*,\mathrm{end}}$ of a task ($o_{h,i}^{*}$) at any level ${}^{*}$ is the estimated start time $t_{h,i+1}^{*,\mathrm{start}}$ of the following task ($o_{h,i+1}^{*}$) of the same level *.

**Figure 4:**
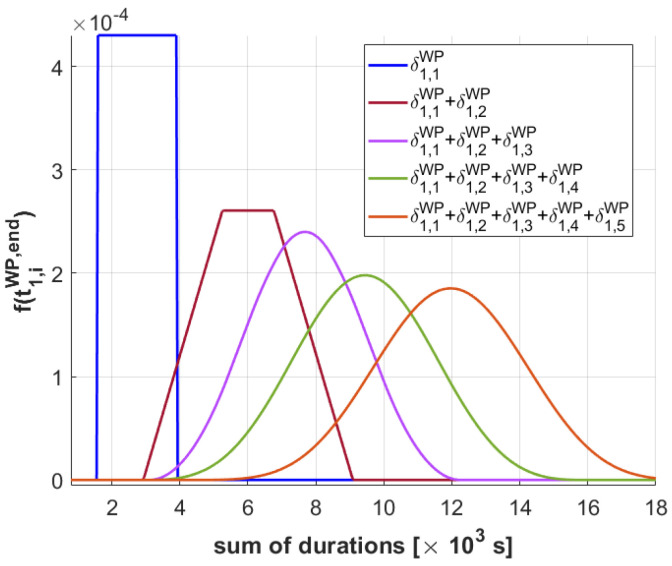
Probability distribution of the sum of durations of tasks, which are uniform distributed random variables of ($m_{\delta_{h,i}^{\mathrm{WP}}},u_{\delta_{h,i}^{\mathrm{WP}}}$): (3132, 791), (4368, 1012), (2876, 698), (3856, 971), (4112, 1263).

To understand better the implications of the previous distributions, we represent in Figure 4 the results of Equation (4) when considering up to five $\delta_{h,i}^{\mathrm{WP}}$ random variables with the median $m_{\delta_{h,i}^{\mathrm{WP}}}$ and half length $u_{\delta_{h,i}^{\mathrm{WP}}}$ provided at the figure caption. We can observe how the sum of more than two uniform distributed random variables assimilates towards a Gaussian distribution, while the variance grows with the number of random variables involved in the sum. This implies that the distribution becomes more wide-spread, and in our case, that the knowledge on the start or end time of a task further in the future is more “uncertain” than the knowledge on the start or end time of a task in the near future. Besides, it is possible to calculate the minimum and maximum values of $t_{h,i}^{*,\mathrm{end}}$, as the density functions calculated with Equation (4) have a limited support. Finally, it is worth noting that a correct estimation of the probability distribution of the sum of tasks durations implies the correct estimation of the start or end time of the tasks at each level. This is essential, especially at the MA-level, since some of the mission requirements and mission constraints stated in Section 2.4 and Section 2.5 depend on the time-varying weather conditions and on the time-dependent requirements, associated to the mission time windows used to decide if the high-level tasks can be rewarded.

### 3.2. Objectives

The aim of this work is to present a multi-HAPS planner that optimizes the HAPSs tasks plans, whose joint quality is evaluated by the three objective functions defined in the following subsections, each contributing to a different aspect of the overall operational performance.

#### 3.2.1. Expected Cumulative Rewards per Hour

This objective focuses on the reward the team of HAPS can gain with the generated plan. Since the success of a task depends on its execution time (i.e., on its start and end time), which can only be probabilistically estimated using Equation (4), the reward can only be estimated with an expected cumulative reward function.

To do it, we exploit the Time-Dependent Markov Decision Process (TiMDP) of Boyan and Littman [17] to calculate the expected cumulative reward, obtained when applying at state $s_{h,i}$ (which in our case contains, among others, the current location of the HAPS) and at time $t_{i}$ the remaining plan ${\tilde{\pi }}_{h,i:n}^{\mathrm{MA}}$ under the weather $w_{t_{i}}$ forecasted for $t_{i}$:

(7) $$\begin{array}{cc} E(R|s_{h,i},t_{i} & \;\;\;\;,{\tilde{\pi }}_{h,i:n}^{\mathrm{MA}},w_{t_{i}})=\Sigma_{\mu \in \{\mathrm{succ},\mathrm{fail}\}}L\left(\mu |s_{h,i},t_{i},o_{h,i}^{\mathrm{MA}},w_{t_{i}}\right)\cdot \\ \; & \cdot \int_{R}f\left(t_{h,i}^{\mathrm{MA},\mathrm{end}}=t_{i+1}\right)\cdot \left[R\left(\mu ,o_{h,i}^{\mathrm{MA}},t_{i+1}\right)+E\left(R|s_{h,i+1},t_{i+1},{\tilde{\pi }}_{h,i+1:n}^{\mathrm{MA}},w_{t_{i+1}}\right)\right]dt_{i+1}, \end{array}$$

where $L(\mu |s_{h,i},t_{i},o_{h,i}^{\mathrm{MA}},w_{t_{i}})$ is the likelihood that action $o_{h,i}^{\mathrm{MA}}$, performed at time $t_{i}$ at state $s_{h,i}$ is successful (μ = succ) or not (μ = fail) under the weather conditions $w_{t_{i}}$ at $t_{i}$; $f(t_{h,i}^{\mathrm{MA},\mathrm{end}}=t_{i+1})$ is the probability density function of ending $o_{h,i}^{\mathrm{MA}}$ at $t_{i+1}$, and $R(\mu ,o_{h,i}^{\mathrm{MA}},t_{i+1})$ is the immediate reward obtained when performing $o_{h,i}^{\mathrm{MA}}$ at time $t_{i+1}$ successfully (μ = succ) or unsuccessfully (μ = fail). In the latter case, $R(\mu =\mathrm{fail},o_{h,i}^{\mathrm{MA}},t_{i+1})=0$.

Although Equation (7) was originally designed to devise a strategy aiming at optimizing the success rate of arriving in time at a destination using different combinations of means of transport, we do not seek to use TiMDP this way. Rather, we exploit the equation as a model for computing the expected cumulative reward of a complete plan $E(R|s_{h,0},t_{0},\pi_{h}^{\mathrm{MA}},w_{t_{0}})$, which can be done using a backward iteration, since the immediate reward $R(\mu ,o_{h,i}^{\mathrm{MA}},t_{i+1})$ is piecewise constant with respect to $t_{i+1}$. Moreover, due to the piecewise constant weather data, $E(R|s_{h,i},t_{i},{\tilde{\pi }}_{h,i:n}^{\mathrm{MA}},w_{t_{i}})$ is piecewise constant too. Therefore, the integration can be performed in piecewise time intervals that are generated using the minimum and maximum of the start time of a task (according to $f(t_{h,i}^{\mathrm{MA},\mathrm{end}}$), as well as the minimum and maximum bounding times of the piecewise constant coverage.

Exploiting the formulation in Equation (7) for computing the expected cumulative reward also leverages the following:

- It takes into account the immediate reward $R(\mu ,o_{h,i}^{\mathrm{MA}},t_{i+1})$ obtained after monitoring the selected mission area at end time $t_{i+1}$, as well as the reward of the remaining action plan ${\tilde{\pi }}_{h,i+1:n}^{\mathrm{MA}}$.
- It considers the likelihood of performing the task successfully and unsuccessfully, depending on the weather conditions, or more specifically, on the cloud coverage, which is related to the mission requirements (i.e., MR1) listed in Section 2.4.
- It exploits the weighting imposed by $f(t_{h,i}^{\mathrm{MA},\mathrm{end}}=t_{i+1})$ at the given times $t_{i+1}$. This is helpful since the weather forecast is constantly updated and a replanning can occur in the future. Therefore, while it is important to “look forward” in the plan to optimize it for a longer time horizon, we allocate more weighting according to immediacy, since a replanning could be triggered to improve the plan quality in the future.

Finally, since multiple HAPS can be involved and the start time of the plan for each HAPS can be different, we accumulate the expected reward of each HAPS to obtain the Objective Function (OF) of the expected cumulative reward $OF_{\mathrm{rew}}$:

(8) $$OF_{\mathrm{rew}}\left(\pi^{\mathrm{MA}}\right)=\Sigma_{h}E\left(R|s_{h,0},t_{h,0},\pi_{h}^{\mathrm{MA}},w_{t_{0}}\right).$$

#### 3.2.2. Effort

Although the mission rewards are important, they are not the only objective to consider. Global client satisfaction must be taken into account too. That is, to keep the clientele satisfied, the HAPS team is required to perform monitoring tasks for as much of their time in the air as possible. Therefore, we consider the objective function of effort, which is the percentage of time spent on monitoring the LOIs:

(9) $$OF_{\mathrm{eff}}\left(\pi^{\mathrm{LOI}}\right)=\frac{\Sigma_{h}\Sigma_{l}E\left(\delta_{h,l}^{\mathrm{LOI}}\right)*\mathrm{isMonitor}\left(o_{h,l}^{\mathrm{LOI}}\right)}{T_{h}^{\max}-t_{h,0}}=\frac{\Sigma_{h}\Sigma_{l}m_{\delta_{h,l}^{\mathrm{LOI}}}*\mathrm{isMonitor}\left(o_{h,l}^{\mathrm{LOI}}\right)}{T_{h}^{\max}-t_{h,0}},$$

where $T_{h}^{\max}$ is the end time of the plan horizon set for HAPS *h*, $E(\delta_{h,l}^{\mathrm{LOI}})$ is the expected duration of the monitoring task for $o_{h,l}^{\mathrm{LOI}}$ which, given the symmetric distribution of the random variable, is the median duration $m_{\delta_{h,l}^{\mathrm{LOI}}}$, and $\mathrm{isMonitor}(o_{h,l}^{\mathrm{LOI}})$ returns 1 if the action $o_{h,l}^{\mathrm{LOI}}$ consists of monitoring a LOI (i.e., if $o_{h,l}^{\mathrm{LOI}}$ equals ${\mathrm{monitor}}_{\mathrm{LOI}\#\in \mathrm{MA}\#}$) and 0 otherwise.

This objective function contributes to preventing the HAPS from trying too hard to reach more rewarding MAs by crossing multiple corridors and WAs.

#### 3.2.3. Diversity

In the presence of missions that are much more rewarding than others, the plan computation can be extremely unfavorable for less rewarding missions. This has a long-term negative effect to the HAPS team in regard of “customer service”. In order to satisfy a more diverse clientele pool, the diversity objective function $OF_{\mathrm{div}}$ is devised using the Simpson index [18]:

(10) $$OF_{\mathrm{div}}\left(\pi^{\mathrm{MA}}\right)=1-\frac{\Sigma_{c=1}^{N_{\mathrm{MA}}}n_{c}\left(n_{c}-1\right)}{N(N-1)},$$

where $N_{\mathrm{MA}}$ is the total number of MAs (or clients) considered in the mission scenario, $n_{c}$ is the number of occurrences of ${\mathrm{MA}}_{c}$ in the task plan, and *N* is the total number of MAs within the task plan. Note that the function only considers what happens with the mission areas, ignoring what is occurring in the waiting areas.

Optimizing this objective reduces the probability of drawing the same MA when two of them are drawn without replacement from a given plan, preventing therefore the bias towards rewarding missions.

### 3.3. Constraints

While the missions’ requirements presented in Section 2.4 are considered in the evaluation of the objective function $OF_{\mathrm{rew}}$, the mission constraints presented in Section 2.5 are evaluated with different constraint criteria.

Besides, while **MC5** and **MC6** are directly encoded in the solutions manipulated by the EA-based planner described in Section 4.1 (and hence, they are never violated), the remaining criteria (**MC1**–**MC4**) are evaluated with the functions described in Section 3.3.1, Section 3.3.2 and Section 3.3.3.

Finally, it is worth noting that our constraint functions consider the number of times that each criterion is violated. Detailed information of this way of proceeding is presented in Section 3.3.4.

#### 3.3.1. Safety

The safety constraint criterion comprises **MC1** and **MC2** and is violated if the MA# is a risk zone (due either to substantial obstacle occlusion or strong wind) while HAPS *h* is operating in it. Since the position of a HAPS is probabilistic due to the uncertainty in the task durations (as Equation (4) states), the constraint function associated to the safety violation $\varphi_{\mathrm{saf}}\left(\pi^{\mathrm{MA}}\right)$ is incremented if the probability of operating any HAPS *h* in a MA representing a risk zone is greater than a predefined threshold $p_{\mathrm{saf}}$:

(11) $$P\left(\left[t_{h,i}^{\mathrm{MA},\mathrm{start}},t_{h,i}^{\mathrm{MA},\mathrm{end}}\right]\cap T_{\mathrm{risk}}\left(o_{h,i}^{\mathrm{MA}}\right)\neq \emptyset \right)>p_{\mathrm{saf}},$$

where $t_{h,i}^{\mathrm{MA},\mathrm{start}}$ and $t_{h,i}^{\mathrm{MA},\mathrm{end}}$ are the start and end time of HAPS *h* performing the monitoring task $o_{h,i}^{\mathrm{MA}}$, while $T_{\mathrm{risk}}\left(o_{h,i}^{\mathrm{MA}}=\mathrm{MA}\#\right)$ is the set of time windows where the MA# associated to $o_{h,i}^{\mathrm{MA}}$ represents a risk zone. Alternatively, we can compute the same $\varphi_{\mathrm{saf}}\left(\pi^{\mathrm{MA}}\right)$ by incrementing its value if

(12) $$\exists t\in \left[\min\left(t_{h,i}^{\mathrm{MA},\mathrm{start}}\right),\max\left(t_{h,i}^{\mathrm{MA},\mathrm{end}}\right)\right]\cap T_{\mathrm{risk}}\left(o_{h,i}^{\mathrm{MA}}\right),\;P\left({\mathrm{pos}}_{h}\left(t\right)\in o_{h,i}^{\mathrm{MA}}\right)>p_{\mathrm{saf}},$$

where *t* belongs to the intersection of $T_{\mathrm{risk}}\left(o_{h,i}^{\mathrm{MA}}\right)$ with the maximum time span that the HAPS could be performing task $o_{h,i}^{\mathrm{MA}}$, ${\mathrm{pos}}_{h}\left(t\right)$ is the position of HAPS *h* at time *t*, and ${\mathrm{pos}}_{h}\left(t\right)\in o_{h,i}^{\mathrm{MA}}$ indicates that HAPS *h* is positioned within the MA in which the monitoring task $o_{h,i}^{\mathrm{MA}}$ takes place. The probability $P({\mathrm{pos}}_{h}\left(t\right)\in o_{h,m}^{\mathrm{MA}})$ in Equation (12) can be further simplified as Equation (13) states, by taking advantage, in the second last step, of the fact that $P\left(t<t_{h,i}^{\mathrm{MA},\mathrm{start}}\;\cap \;t>t_{h,i}^{\mathrm{MA},\mathrm{end}}\right)=P\left(t<t_{h,i}^{\mathrm{MA},\mathrm{start}}\;\cap \;t>t_{h,i}^{\mathrm{MA},\mathrm{start}}+\delta_{h,i}^{\mathrm{MA}}\right)\overset{\delta_{h,i}^{\mathrm{MA}}>0}{=}0.$

(13) $$\begin{array}{cc} P(\;\;\;\; & \;\;{\mathrm{pos}}_{h}\left(t\right)\in o_{h,i}^{\mathrm{MA}})=\\ = & P(t_{h,i}^{\mathrm{MA},\mathrm{start}}<t<t_{h,i}^{\mathrm{MA},\mathrm{end}})\\ = & \;P\left(t>t_{h,i}^{\mathrm{MA},\mathrm{start}}\right)+P\left(t<t_{h,i}^{\mathrm{MA},\mathrm{end}}\right)-P\left(t>t_{h,i}^{\mathrm{MA},\mathrm{start}}\;\cup \;t<t_{h,i}^{\mathrm{MA},\mathrm{end}}\right)\\ = & \;P\left(t>t_{h,i}^{\mathrm{MA},\mathrm{start}}\right)+\left[1-P\left(t>t_{h,i}^{\mathrm{MA},\mathrm{end}}\right)\right]-\left[1-P\left(t<t_{h,i}^{\mathrm{MA},\mathrm{start}}\;\cap \;t>t_{h,i}^{\mathrm{MA},\mathrm{end}}\right)\right]\\ = & \;P\left(t>t_{h,i}^{\mathrm{MA},\mathrm{start}}\right)-P\left(t>t_{h,i}^{\mathrm{MA},\mathrm{end}}\right)\\ = & \left\{\begin{array}{cc} 0, & \mathrm{if}\;\;\;t<\min(t_{h,i}^{\mathrm{MA},\mathrm{start}})\;\\ \int_{\min(t_{h,i}^{\mathrm{MA},\mathrm{start}})}^{t}f\left(t_{h,i}^{\mathrm{MA},\mathrm{start}}=t\right)dt, & \mathrm{if}\;\;\;t<\min(t_{h,i}^{\mathrm{MA},\mathrm{end}})\\ \int_{\min(t_{h,i}^{\mathrm{MA},\mathrm{start}})}^{t}f\left(t_{h,i}^{\mathrm{MA},\mathrm{start}}=t\right)dt-\int_{\min(t_{h,i}^{\mathrm{MA},\mathrm{end}})}^{t}f\left(t_{h,i}^{\mathrm{MA},\mathrm{end}}=t\right)dt, & \mathrm{otherwise}. \end{array}\right. \end{array}$$

#### 3.3.2. Coexistence

**MC3** is violated and its corresponding violation index $\varphi_{\mathrm{coex}}\left(\pi^{\mathrm{MA}}\right)$ is incremented by 1, if the probability of two HAPSs (*h* and $h^{\prime }$, with $h\neq h^{\prime }$) operating at the same time *t* in the same MA# (i.e., $o_{h,i}^{\mathrm{MA}}=o_{h^{\prime },j}^{\mathrm{MA}}=\mathrm{MA}\#$) is greater than an imposed threshold $p_{\mathrm{coex}}$. That is, if

(14) $$\exists t\in \left[\min\left(t_{h,i}^{\mathrm{MA},\mathrm{start}}\right),\max\left(t_{h,i}^{\mathrm{MA},\mathrm{end}}\right)\right],\;P\left({\mathrm{pos}}_{h}\left(t\right)\in o_{h,i}^{\mathrm{MA}}\;\cap \;{\mathrm{pos}}_{h^{\prime }}\left(t\right)\in o_{h^{\prime },j}^{\mathrm{MA}}\right)>p_{\mathrm{coex}}.$$

The probability in the previous expression can be expressed as the product of two probabilities, each of them computable using Equation (13):

(15) $$P\left({\mathrm{pos}}_{h}\left(t\right)\in o_{h,i}^{\mathrm{MA}}\;\cap \;{\mathrm{pos}}_{h^{\prime }}\left(t\right)\in o_{h^{\prime },j}^{\mathrm{MA}}\right)=P\left({\mathrm{pos}}_{h}\left(t\right)\in o_{h,i}^{\mathrm{MA}}\right)\cdot P\left({\mathrm{pos}}_{h^{\prime }}\left(t\right)\in o_{h^{\prime },j}^{\mathrm{MA}}\right).$$

**Figure 5:**
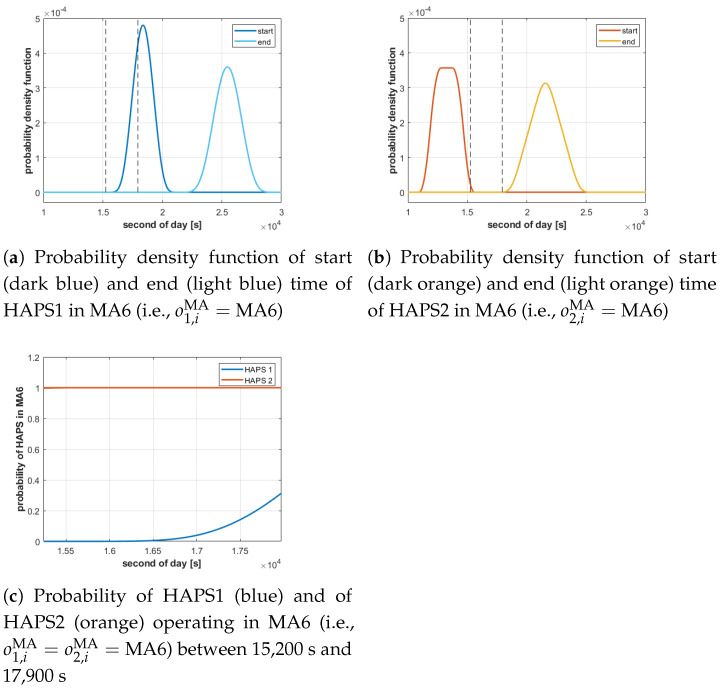
Probabilistic evaluation of the start and end time of the operation of HAPS in a MA and probability of their operation in the MA within the duration marked by the vertical dash lines.

To illustrate how the coexistence constraint is evaluated, Figure 5 shows the probabilistic evaluation of the existence of two HAPSs in MA6 for a particular $\pi^{\mathrm{MA}}$. Given the probability density functions of the start and end time of each HAPS in MA6, obtained with Equations (4) and (5), and represented in Figure 5a,b, the probabilities of the presence of each HAPS in MA6 (i.e., $P({\mathrm{pos}}_{h}\left(t\right)\in o_{h,i}^{\mathrm{MA}})$ and $P({\mathrm{pos}}_{h^{\prime }}\left(t\right)\in o_{h^{\prime },j}^{\mathrm{MA}})$ with $o_{h,i}^{\mathrm{MA}}=o_{h^{\prime },j}^{\mathrm{MA}}=\mathrm{MA}6$) are estimated with Equation (13) and displayed in Figure 5c. Besides, for clarity of the representation, the time limits of Figure 5c are marked with vertical dashed lines in Figure 5a,b. The constraint function $\varphi_{\mathrm{coex}}\left(\pi^{\mathrm{MA}}\right)$ associated to the coexistence of both HAPSs in the MA will be incremented if the product of the two probabilities represented in Figure 5c (i.e., $P\left({\mathrm{pos}}_{h}\left(t\right)\in o_{h,i}^{\mathrm{MA}}\right)\cdot P\left({\mathrm{pos}}_{h^{\prime }}\left(t\right)\in o_{h^{\prime },j}^{\mathrm{MA}}\right)$) exceeds the threshold $p_{\mathrm{coex}}$.

#### 3.3.3. Connection

This constraint considers the connectivity of mission elements of a plan represented at the MA-level. The mission elements (i.e., either MA# or WA#) are connected if and only if there is a corridor connecting two consecutive elements in the MA-level plan. Each lack of connection increments the constraint criterion $\varphi_{\mathrm{con}}\left(\pi^{\mathrm{MA}}\right)$ by 1.

#### 3.3.4. Overall Constraint Violation

As each constraint violation increments $\varphi_{\mathrm{criteria}}\left(\pi^{\mathrm{MA}}\right)$ of its corresponding criteria; a non-null $\varphi_{\mathrm{criteria}}\left(\pi^{\mathrm{MA}}\right)$ implies the infeasibility of the plan. For that reason, the overall constraint function of a given plan $\pi^{\mathrm{MA}}$ is simply the sum of all the constraint criteria:

(16) $$\varphi \left(\pi^{\mathrm{MA}}\right)=\varphi_{\mathrm{saf}}\left(\pi^{\mathrm{MA}}\right)+\varphi_{\mathrm{coex}}\left(\pi^{\mathrm{MA}}\right)+\varphi_{\mathrm{con}}\left(\pi^{\mathrm{MA}}\right).$$

Finally, note that in order to determine during the evaluation of $\varphi_{\mathrm{saf}}\left(\pi^{\mathrm{MA}}\right)$ and $\varphi_{\mathrm{coex}}\left(\pi^{\mathrm{MA}}\right)$ if there is a *t* where Equations (12) and (14) hold, the time variable *t* is discretized, within the corresponding intervals given in those equations, into equally spaced time instances.

## 4. Implementation of a GA-Guided Hierarchical Task Planner

The purpose of the planner presented in this section is to perform the task planning for a group of HAPSs that maximize the objective functions presented in Section 3.2 (i.e., reward, effort, and diversity), while ensuring that it is feasible according to the constraint criteria introduced in Section 3.3 (i.e., safety, coexistence, and connection).

To achieve it, we use the Genetic Algorithm (GA) based planner described in Section 4.3 that manipulates the codification of the solutions presented in Section 4.1, which encodes the sequence of MA-level tasks that determines the (sub)optimal temporal hierarchical decomposition of tasks governed by the approach presented in Section 4.2.

### 4.1. Plan Codification

The solutions that the planner must provide are hierarchical plans ($\pi^{\mathrm{MA}},\pi^{\mathrm{LOI}},\pi^{\mathrm{WP}}$) to be presented as suggestions to the HAPS operator during the monitoring mission. Each plan $\pi^{*}$ in the hierarchy, as its formal description in Section 3.1 shows, consists of a list of tasks and their start times and durations. However, as the latter are affected by the weather conditions, we decide to code only the tasks in the optimizer and estimate their timing, when required, in the evaluation of the objective and constraint functions.

Besides, a hierarchical plan decomposes the tasks at a given level into a set of tasks of a lower level, until the set of primitive tasks, or rather “actions”, are obtained. In our case, the decomposition into tasks at the intermediate (LOI) level and at the lower (WP) level are given by a fixed set of rules. In particular, a $o_{h,i}^{\mathrm{MA}}=\mathrm{WA}\#$ task is directly converted into $o_{h,j}^{\mathrm{LOI}}=$ fly${}_{\mathrm{WA}\#}$, while a $o_{h,i}^{\mathrm{MA}}=\mathrm{MA}\#$ is decomposed into the sequence of $o_{h,j}^{\mathrm{LOI}}=$ monitor${}_{\mathrm{LOI}\#\in \mathrm{MA}\#}$ tasks that implies the sequential monitoring of all the LOIs (without revisit) in the MA before departing. As the number of possible sequences of LOIs in a MA is the number of their permutations, we fix the order in which the LOIs are visited, starting with the LOI closest to the HAPS entry point in the MA and following the order that minimizes the distance of the HAPS within the MA. This way of proceeding ensures the shortest travel distance within a MA, simplifies the optimization problem and accelerates the computation of the plans, as we can precalculate all the orders for a given MA, since we know beforehand all its possible entry and exit points. Besides, it is justified by the fact that the weather conditions do not vary much within a MA. Finally, the decomposition of LOI actions in waypoint actions is usually straightforward and the only possible choices are also fixed. As Figure 2a depicts, this can be done by connecting the entry and exit points of a corridor, the entry point at a MA to the start point of the scan, followed by the points that mark the start and end of a scan track and finally, the exit point of the MA.

Taking into account the previous ideas, the remaining effort to determine the (sub)optimal solution lies in the search for the optimal lists of high level tasks (i.e., MA# and WA#) that each HAPS must perform. As the number of possible WA and MA is finite, the elements of the lists can also be encoded with a finite alphabet of labels. Hence, for the GA-based planner, the solution will be encoded as an array of as many elements as HAPSs, where each element contains the list of the high level tasks (MA# and WA#) of each HAPS. Finally, to distinguish this encoding from the corresponding hierarchical plan ($\pi^{\mathrm{MA}},\pi^{\mathrm{LOI}},\pi^{\mathrm{WP}}$), we represent ${\mathrm{sol}}_{k}$ as the *k*-th possible solution of the planner, $sol_{k}\left[h\right]$ as the part of the solutions for HAPS *h*, and $sol_{k}\left[h\right]\left[i\right]$ as the i−th task at the MA-level (i.e., MA# or WA#) to be performed by HAPS *h* of the *k*-th solution of the planner.

The next section explains how to obtain a hierarchical plan ($\pi_{h}^{\mathrm{MA}},\pi_{h}^{\mathrm{LOI}},\pi_{h}^{\mathrm{WP}}$) from a given $sol_{k}\left[h\right]$.

### 4.2. Temporal Hierarchical Task Decomposition

Since the coding of the solutions manipulated by the GA that supports the search of (sub)optimal solutions in our planner is only a sequence of MA# and WA# actions, and the objective and constraint functions used to evaluate them require a hierarchical plan and the estimated end time of the tasks at different levels, in this section we detail, with the help of the pseudo-code presented in Algorithm 1, how the conversion from $sol_{k}\left[h\right]$ to $\pi_{h}$ is carried out.

The algorithm inputs are the solution plan $sol_{k}\left[h\right]$ that encodes only the tasks at the MA level and the start mission time $t_{h}^{0}$ for HAPS *h*, and its output is the hierarchical plan $\pi_{h}=\left(\pi_{h}^{\mathrm{MA}},\pi_{h}^{\mathrm{LOI}},\pi_{h}^{\mathrm{WP}}\right)$. To start with, Line 1 initializes the hierarchical plan as empty sequences, while Line 2 initializes an empty list for the limits of the duration of each WP task (which will be used later to estimate the density functions of the end time of the tasks at WP level) and Line 3 initializes the Boolean flag $b_{\mathrm{finish}}$ (which is meant to keep track of the temporal plan length and ignore the tasks that start after the maximum plan horizon $T_{h}^{\max}$ has been reached).

After the initialization steps, three nested loops are implemented, in order to be able to decompose tasks at MA-level into primitive tasks at WP-level and to determine the probability distributions of the end time of the tasks of the highest levels from the primitive tasks of the lowest. As the number of nested loops depends on the depth of the decomposition, in our case, three loops are necessary, since the primitive tasks (at WP-level) lie two levels below the MA-level at which the initial $sol_{k}\left[h\right]$ is given.

The particular behavior implemented in the three loops is the following. At Line 7 the current MA task in $sol_{k}\left[h\right]\left[i\right]$ is selected to be decomposed into an ordered list of LOI tasks at Line 8. Next, at Line 9, temporary partial plans of the lower level tasks (${\tilde{\pi }}_{h}^{\mathrm{LOI}}$ and ${\tilde{\pi }}_{h}^{\mathrm{WP}}$) are initialized as empty lists to be able to temporarily store the sequences of tasks obtained after the decomposition of the selected task $o_{\mathrm{current}}^{\mathrm{MA}}$ at the MA-level into the lists of tasks at the LOI-level or WP-level. This lowest level decomposition into primitive tasks happens at Lines 13 and 14, where the current LOI task $o_{\mathrm{current}}^{\mathrm{LOI}}$ is selected and decomposed into the corresponding list of WP tasks. Next, we start processing sequentially each of the primitive tasks $o_{\mathrm{current}}^{\mathrm{WP}}$ of our hierarchical task plan. For this, at Line 19 we determine (using Equation (3)) the limits (minimum and maximum) of the duration needed for the task and append them in Line 20 to the list of limits list_limits. Next, at Line 21 the minimum temporal plan length up to the current $o_{\mathrm{current}}^{\mathrm{WP}}$ is checked to see if the plan horizon $T_{h}^{\max}$ is exceeded. If that is the case, the decomposition must stop and all lower-level partial plans should not be accounted for. If $T_{h}^{\max}$ is not exceeded, at Line 26 the probability distribution on the end time of task $o_{\mathrm{current}}^{\mathrm{WP}}$ is determined with Equation (4), and at Line 27 the current $o_{\mathrm{current}}^{\mathrm{WP}}$ task and the determined probability distribution is appended to the temporal plan ${\tilde{\pi }}_{h}^{\mathrm{WP}}$. Next and after looping over all the tasks at the WP-level (if the finishing time has not been reached) the probability distribution $f(t_{\mathrm{current}}^{\mathrm{end},\mathrm{LOI}})$ of the end time of the current LOI-level task is assigned the probability distribution $f(t_{\mathrm{current}}^{\mathrm{end},\mathrm{WP}})$ of the end time of the last WP-level task, and $o_{\mathrm{current}}^{\mathrm{LOI}}$ and $f(t_{\mathrm{current}}^{\mathrm{end},\mathrm{WP}})$ are appended to the temporal plan ${\tilde{\pi }}_{h}^{\mathrm{LOI}}$. Next, a similar process is repeated to obtain at Line 38 the probability of the end time of the current action of the MA level plan from the probability distribution of the end time of the last LOI level action and to update at Line 39 the MA plan $\pi_{h}^{\mathrm{MA}}$. Finally, at Lines 40 and 41 the partial plans, ${\tilde{\pi }}_{h}^{\mathrm{LOI}}$ and ${\tilde{\pi }}_{h}^{\mathrm{WP}}$ are appended to their corresponding plans $\pi_{h}^{\mathrm{LOI}}$ and $\pi_{h}^{\mathrm{WP}}$.
**Algorithm 1:** Temporal hierarchical task decomposition
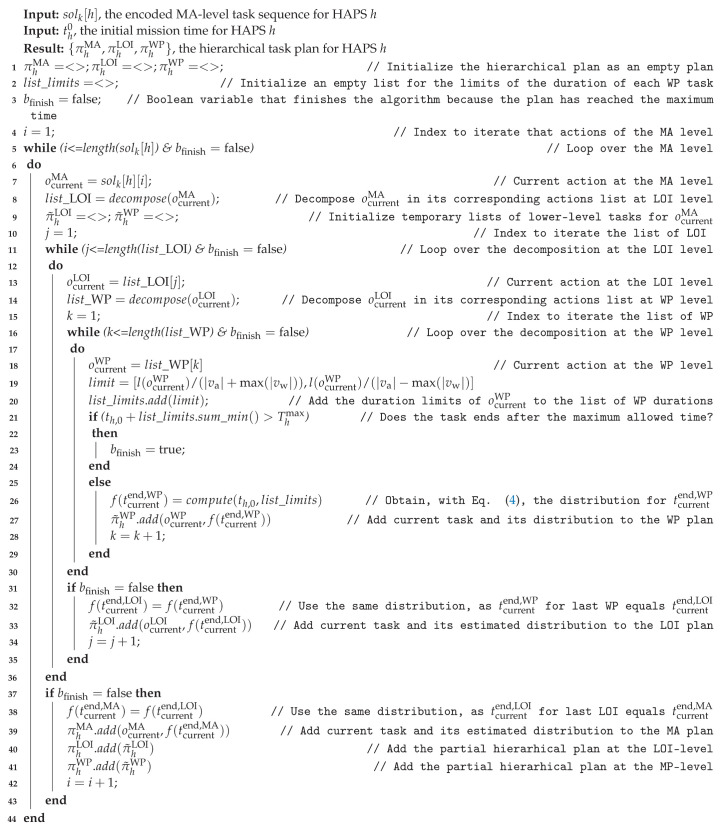


### 4.3. GA-Guided Search of the Best Plans

Algorithm 1 determines the hierarchical plan associated to a given HAPS *h* and list of MA-level tasks $sol_{k}\left[h\right]$. However, determining the best list of MA-level tasks ${\mathrm{sol}}_{k}$ for all the HAPSs in the mission is extremely complex, as we are facing a probabilistic time-dependent multiple-vehicle routing problem, where multiple objective functions ($OF_{\mathrm{rew}}$, $OF_{\mathrm{div}}$, and $OF_{\mathrm{div}}$) and constraint criteria ($\varphi_{\mathrm{saf}}$, $\varphi_{\mathrm{coex}}$ and $\varphi_{\mathrm{con}}$) have to be considered. To tackle it, we develop a mission planner that exploits the Non-dominated Sorting Genetic Algorithm (NSGA-II, Ref. [19]) to look for the optimal ${\mathrm{sol}}_{k}$. For the clarity and completeness of the paper, our implementation of NSGA-II is recapitulated in Algorithm 2, along with the specifics relevant to this work.
**Algorithm 2:** NSGA-II-guided search of nondominated solutions of hierarchical plans
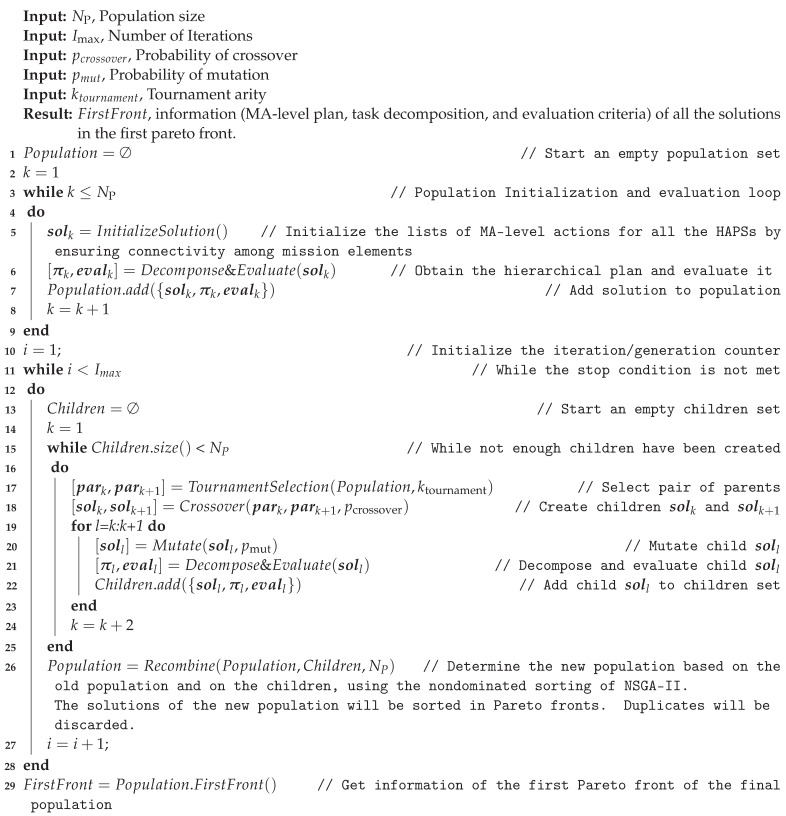


Between Lines 1 and 9, Algorithm 2 performs the initialization steps, consisting of generating $N_{p}$ solutions of high-level lists of actions (${\mathrm{sol}}_{k}$), performing their decompositions into hierarchical plans (π), and evaluating their objective functions and constraint criteria (${\mathrm{eval}}_{k}=\left[OF_{\mathrm{rew}},OF_{\mathrm{div}},OF_{\mathrm{div}},\varphi_{\mathrm{saf}},\varphi_{\mathrm{coex}},\varphi_{\mathrm{con}}\right]$). To do it, on the one hand, InitializeSolution() generates a population of solution plans that fulfill the connectivity constraint, by appending to $sol_{k}\left[h\right]\left[i+1\right]$ a mission element (MA# or WA#) randomly selected (according to a uniform distribution) among the mission elements connected to the last one $sol_{k}\left[h\right]\left[i\right]$. Besides, a predetermined minimum duration, used to decide how long the initialization of ${\mathrm{sol}}_{k}$ should take for each mission, is calculated based on the maximum ground speed ($v_{a}+\left|\max\left(v_{w}\right)\right|$) and on a travel distance that lower-bounds all realistic travel distances for the mission element derived from the tasks at the WP-level (i.e., the shortest diagonal distance of the mission element). On the other hand, $Decompose\&Evaluate({\mathrm{sol}}_{k})$ performs the decomposition into hierarchical plans of all the lists of high level actions ${\mathrm{sol}}_{k}\left[h\right]$ using Algorithm 1 and evaluates the obtained plans $\pi_{k}$ using the objective functions and constraint criteria. Finally, the Population is formed by the high-level list of actions ${\mathrm{sol}}_{k}$, their corresponding hierarchical plan decomposition $\pi_{k}$, and their corresponding evaluation ${\mathrm{eval}}_{k}$.

Next, the generation loop of the algorithm is performed, between Lines 11 and 28, until reaching the stop condition, consistent on testing if a predefined number of iterations is met. In each generation (algorithm iteration), the new set of solutions, named Children in Algorithm 2, are created by selecting from Population pairs of solution plans expressed at the MA-level (named ${\mathrm{par}}_{k}$ and ${\mathrm{par}}_{k+1}$), which will undergo crossover, mutation, decomposition, and evaluation (see Lines 18 to 21). Afterwards, the old and the new population are combined in Line 26 to determine the new population of the following generation.

In particular, the pairs of parents selection is performed with TournamentSelection$\left(Population,k_{\mathrm{tournament}}\right)$ that implements the *k*-tournament operator proposed in [19] for constrained multiobjective problems. That is, for each parent, it selects randomly, according to the uniform distribution, $k_{\mathrm{tournament}}$ solutions of Population, and among them it selects the best one, preferring infeasible solutions with smaller φ(π) to infeasible solutions with a bigger φ(π), feasible solutions (i.e., those with φ(π)=0) to infeasible ones (i.e., those with φ(π)>0) and the Pareto dominating feasible solutions to the dominated ones.

**Figure 6:**
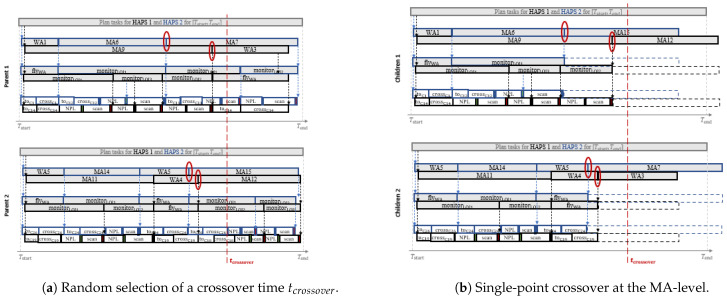
Single-point crossover with a random selection of the temporal crossover point.

Next, the crossover of the two parents (${\mathrm{par}}_{k}$ and ${\mathrm{par}}_{k+1}$) is performed with Crossover$\left({\mathrm{par}}_{k},{\mathrm{par}}_{k+1},p_{crossover}\right)$, that implements a single-point crossover that takes into consideration the expected ending time of the MA-level tasks of each parent. That is, unlike the typical genetic operators for crossover (which select the gene where the crossover should be performed in both parents), we select randomly, according to the uniform distribution and as shown in Figure 6a, the crossover time $t_{\mathrm{crossover}}$. Each parent is then divided into a head and tail component at the start time of a task (at the MA-level) closest to $t_{crossover}$ (as marked in the red ellipses), and afterwards the head of one parent and the tail of the other (and vice versa) are concatenated to build the new list of solutions of each child, as shown in Figure 6b. Besides, the probability of crossover $p_{crossover}$ is used to decide, for each pair of parents, if they should undergo the crossover process or if they should be directly copied as new possible solutions.

After crossover, each child is mutated in Line 20 with $Mutate({\mathrm{sol}}_{l},p_{\mathrm{mut}})$, which uses the probability of mutation $p_{\mathrm{mut}}$ to determine, according to the uniform distribution, if each of the MA-tasks in ${\mathrm{sol}}_{l}$ has to mutate and be changed by any other MA# or WA# task randomly selected at the MA-level.

After mutation, each of the children is decomposed and evaluated with Decompose&$Evaluate({\mathrm{sol}}_{k})$. Moreover, as crossover preserves the head actions of already decomposed plans (as Figure 6b shows) and mutation does not influence in the timing (duration) of the parts of the plan that are previous to the mutation point, we can use the corresponding invariant decomposed plans of the parents to perform more efficiently the decomposition of the new children. Besides, it is worth noting that the connection constraint $\varphi_{\mathrm{con}}\left(\pi^{\mathrm{MA}}\right)$ can be violated after a crossover or a mutation. Hence, in order to make the decomposition quicker, the sequence of high level tasks ($<o_{h,i+1}^{\mathrm{MA}},\cdots ,o_{h,end}^{\mathrm{MA}}>$) after the last connected one ($o_{h,i}^{\mathrm{MA}}$) are not decomposed into tasks of lower levels, neither will the density distribution for the ending time of their tasks be determined. Finally, we prefer to use crossover and mutation operators that allow to create unconnected high-level (MA) plans to allow them to be reconnected afterwards, eventually, after other crossovers and mutations. By doing so, the planner can sometimes create invalid solutions that are used by the search process to transverse infeasible regions of the search space while moving from one side of the feasible search space to the other. The planner configurations under analysis in the following section will show the importance of this fact.

Once the children population has been completely created, the Population and their Children are first compared to discard the duplicate solution. Afterwards they are sorted together into nondominated fronts by using the same criteria as in the tournament selection (i.e., their objective functions and constraint criteria are taken into account to prefer feasible to infeasible, solutions that are closer to be feasible to those that are farther to be feasible). Finally, the sorted population is truncated to contain only the best $N_{P}$ solutions, using the crowding distance, as described in [19], to pick the surviving solutions that belong to the last front that can be admitted into the new population.

At the end of the algorithm, once the generation loop has finished, the planner returns the set of solutions that belong to the first front of the last Population. In this front, it is expected to find solutions that fulfill constraints and that are equally good, from the Pareto comparison perspective, regarding the objective functions.

Finally, it is worth noting that although it is not stated in Algorithm 2 for simplicity, all the Population of all iterations obtained by the planner (in the initialization and during the generation loop) are also stored to be able to analyze the performance of the planner, over different scenarios, in the following section.

## 5. Results and Analysis

This section analyzes the performance of the GA-based planner described in this paper for determining the hierarchical task decomposition of a set of HAPSs that carry out realistic monitoring missions in complex time-varying environments with a highly-organized airspace structure. This planner combines the algorithms described in Section 4 as well as the evaluation functions and constraint criteria formally elaborated in Section 3 in order to take into account the realistic mission requirements and constraints described in Section 2.

To highlight the benefits of the planner, different scenarios are used during the performance tests. The subsequent subsections will first introduce the chosen scenarios, followed by a description of the different variants of the planner that are tested (to determine which configurations are better for each scenario), by an interpretation of the graphical representation of the results, and finally, by their in-depth analysis.

The algorithms are implemented in Matlab and tested on a 4-core i7 processor at 1.80 GHz. On average, an iteration takes 15 s and can go up to 30 s under challenging weather conditions or when more HAPSs are involved, due to the constraint evaluations. The computation time is acceptable for the mission at hand, as the planning is meant to be performed prior to the execution (as opposed to real-time planning), and therefore more generous planning time is allowed. Besides, the planner can also be implemented as an “anytime” planner, as the algorithm provides a Pareto front with feasible solutions at each iteration. To accelerate the code in a future release, the evaluation functions could be implemented in C.

### 5.1. Scenarios

The three scenarios considered in the performance tests of this paper share the mission map depicted in Figure 1 and the HAPSs and mission parameters presented in Appendix A. The scenarios differ in the weather data and/or the number of HAPSs involved. The following paragraphs briefly introduce the settings of each scenario, while Table 2 provides an overview of all of them.

*Nominal scenario*. In the first scenario, historical weather data of COSMO-DE (predecessor of COSMO-D2) taken from a relatively calm day in April 2018 is used. The weather conditions are considered moderate, with some strong wind before noon time and some cloudy hours. Besides, the mission is performed by two HAPSs, placed initially at WA2 and WA4, that have to monitor the LOIs depicted in Figure 1 taking into account the rewards and coverage information provided in Table A3. This scenario is useful to see how the planner works under good (nominal) weather conditions.

*Challenging weather scenario*. In this scenario, we use a weather data of the same format as the real weather data considered in the first scenario but synthetically increase the wind to make it stronger. In particular, in some mission areas or corridors, strong wind can occur during more than half of the time of the day. This synthetic scenario is created in order to demonstrate the performance of the planner under challenging weather conditions. Finally, similar to the first scenario, only two HAPSs, placed again initially at WA2 and WA4, are considered in this scenario.

*Three HAPSs scenario*. To demonstrate the scalability of the planner regarding the number of HAPSs, in this scenario the monitoring mission is performed by three HAPSs, placed initially at WA2, WA4, and WA5. The weather conditions are identical to those of the first scenario.

Finally, it is worth noting that the first scenario (labelled as SC1 hereafter) will be considered the basis to compare against the other two, as the second scenario (SC2) is similar to the first but with worsened weather conditions, while the third scenario (SC3) is the first with an additional HAPS.

### 5.2. Planner Configurations

The general input parameters of Algorithm 2 are presented in Table 3. They have been selected after analyzing the behavior of the planner under different combinations of parameters over the presented scenarios.

Besides, several configurations of the GA are considered to optimize the hierarchical task plan and to analyze the performance and benefits of each one for the different scenarios. The three Planner Configurations (PC1, PC2, and PC3) analyzed in the paper are implemented in general according to Algorithm 2 and two of them (PC2 and PC3) contain some slight variations injected into parts of the code to support the following behaviors:

*Planner Configuration 1 (PC1).* The constraint-handling technique proposed by [19] is used for select the pair of parents in the *k*-tournament selection (at Line 17 of Algorithm 2) and for recombining the old and new populations (at Line 26). In other words, solutions that fulfill or are closer to fulfilling the constraints are preferred to solutions that do not fulfill or are further to fulfilling them, and among solutions that are equally good regarding the constraints, solutions that Pareto dominate the others regarding the objective functions are preferred to solutions that are Pareto dominated. As this configuration implements the standard constraint-handling techniques of NSGA-II [19], it is also the one described in Section 4.
*Planner Configuration 2 (PC2).* The constraint-handling criteria are only applied to select the pair of parents in the *k*-tournament selection in Line 17 of Algorithm 2 and ignored during the recombination of old and new populations. That is, during the recombination step in Line 26 of Algorithm 2, the solutions are sorted by only taking into account the ordering imposed by the Pareto comparison of the objective functions. The motivation of this variation is to have a planner configuration that is less “stringent” with the hierarchical plans that violate the constraints (i.e., that have $\varphi (\pi^{\mathrm{MA}})>0$), and to give them more chances to be selected for the next generation (or even be selected as parents for the generation of children solutions of the next iteration).
*Planner Configuration 3 (PC3).* The diversity objective function ($OF_{\mathrm{div}}$) is ignored both during the parents selection and recombination steps. This configuration has been set up to put forth the benefit of considering the diversity (and not only the expected reward or the effort) for planning.

For readers familiar with the stochastic ranking mechanism for constrained evolutionary optimization presented in [20], it is interesting to highlight that PC1 and PC2 represent the two extreme cases that are obtained when the probability of ignoring the constraints is respectively set to 0 (for PC1) or to 1 (for PC2). That is, during the recombination step, in PC1 the constraints are never ignored while in PC2 the constraints are always ignored. Comparing the behavior of the extreme cases will facilitate the understanding of the effects of taking into account (or ignoring) the constraints in the recombination step.

Finally, for the computation of the expected reward using Equation (7), we assume $L(\mu =\mathrm{success}|s_{h,i},t_{i},o_{h,i}^{\mathrm{MA}},w_{t_{i}})=0.8$, if the cloud coverage of $w_{t_{i}}$ is smaller than the image coverage required by the mission, as shown in Table A3. Otherwise, $L(\mu =\mathrm{success}|s_{h,i},t_{i},o_{h,i}^{\mathrm{MA}},w_{t_{i}})=0.2$.

### 5.3. Results Representation

In order to provide an overview on the the weather (wind and cloud coverage) conditions of each scenario, on the time windows where each mission area can be visited, as well as on a representative solution obtained by the planner, we use the graphics displayed in Figures 10–14, whose vertical axes represent, from the bottom to the top, the mission areas (MA#) and waiting areas (WA#), while the horizontal axes represent the hour of the day. Further, the graphics also contain the above-mentioned information on the weather, mission, and plan, which is represented by the following items:

- The light-grey bars represent the time windows with clear sky, while the dark-grey bars signify high cloud coverage above the corresponding MA.
- The light-green bars represent the time windows for the absence of critical weather conditions at the MA or WA, while the dark-green bars signify critical weather conditions for the corresponding MA or WA, e.g., strong wind.
- The light-blue bars represent the time windows where monitoring missions at the corresponding MA are requested (and therefore rewarded), while the dark-blue bars signify the absence of mission request for the corresponding MA.
- Red lines represent the monitoring/fly-by tasks of HAPS1 to be performed on the corresponding MA/WA, according to the representative plan $\pi_{1}^{\mathrm{MA}}$. Moreover, the thicker line in the middle marks the median start and end time, while the thinner lines mark the time range from the minimum starting time to the maximum end time of each task.
- Similarly, blue lines represent the tasks at MA-level to be performed by HAPS2 and magenta lines represent tasks at MA-level to be performed by HAPS3.

Taking into account the previous information, the sequence of mission elements (i.e., MA# and WA#) traversed by each HAPS in a representative solution can be observed, along with the weather conditions and the mission time window of each scenario. For example, in Figure 10a we can observe, following the red line, that HAPS1 moves from WA2 (the starting location of HAPS1, which is not represented in the graphic) to MA1, WA1, WA2, MA2, WA2, WA1, MA3, WA1, and MA3. Besides, MA1 is visited when not requested, while MA2 and the two visits to MA3 are within the correct mission time windows. Besides, MA2 is partially visited under cloudy conditions, which can reduce the expected reward obtained by HAPS1, while MA3 are visited under good weather conditions, which provides HAPS1 two times the total reward of MA3.

Besides, in order to analyze the performance of the different configurations of the planner in different scenarios, we store for each scenario-planner configuration pair and for each iteration of the GA, the values of the objective functions ($OF_{\mathrm{rew}}$, $OF_{\mathrm{eff}}$ and $OF_{\mathrm{div}}$ ) and constraint criteria ($\varphi_{\mathrm{saf}}$, $\varphi_{\mathrm{coex}}$ and $\varphi_{\mathrm{con}}$) of all the feasible solutions (i.e., φ(π)=0) of the best Pareto front obtained during the execution of the algorithm. With that information, we represent the following graphs:

- The evolution over iterations of the Mean and Standard Deviation (M&SD) of the values of each objective function of *the feasible solutions that belong to the best front*. Considering *only feasible solutions of the best front* is initially necessary for the three planner configuration, since it is possible that the first Pareto fronts are initially infeasible. Besides, it is always necessary in PC2, since the fronts are obtained by ignoring the constraints, and therefore, the best front obtained using PC2 can contain infeasible solutions. Moreover, this is meaningful since only the final feasible solution plans of the best Pareto front will be presented to the HAPS operator. The M&SD evolution graphs for each objective function are presented in the first row of Figures 8–13 for Scenario 1, 2, and 3, respectively. The mean and standard deviation values of each objective function are represented in different columns of the figures (left column $OF_{\mathrm{rew}}$, middle column $OF_{\mathrm{eff}}$, and right column $OF_{\mathrm{div}}$). Besides, while the mean is depicted over iterations with a bold line, the shadowed area around it represents the standard deviation, using a different color for each planner configuration (blue for PC1, green for PC2, and red for PC3).
- The evolution of the Maximum (Max) value of each objective function obtained among the solutions of the first Pareto front that also fulfill the constraints are plotted in the lower row of graphs of Figures 8–13. These graphs, organized as the previous and using only a line for the Max value, complement the M&SD evolution graphs as they show the objective values of the best solutions with respect to each objective in the Pareto front.

**Figure 7:**
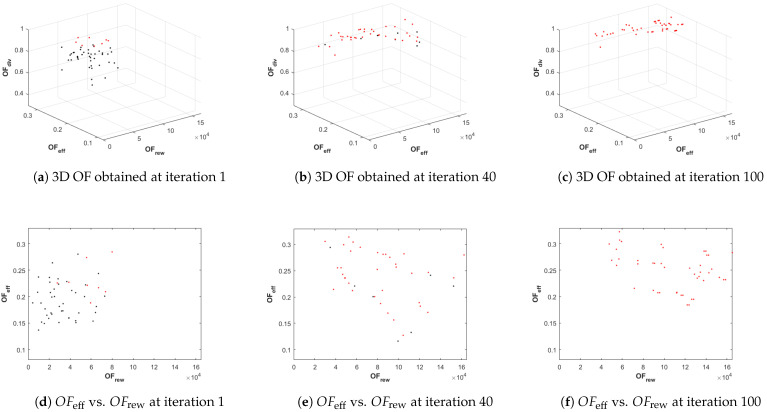
Values of the objective functions of the best Pareto front (marked in red) vs. values of the OFs of the remaining population (marked in black) for SC1 and PC1. The top row of graphics represent in 3D the values of the three OFs, while the second row only shows the values of two of them

Finally, we also use two additional types of graphs in order to analyze further certain scenarios:

- A 3D representation of the values of the three objective functions of all the solutions of the population versus the values of the objective functions of the solutions of the best Pareto front, at selected iterations of the planner (and a 2D representation of $OF_{\mathrm{eff}}$ versus $OF_{\mathrm{rew}}$). This information, represented in Figure 7, marking in red the points associated to the solutions of the best Pareto front and in black the remaining solutions of the population, is used to graphically demonstrate the effectiveness of the planner in evolving and finding solutions of the Pareto front.
- The number of infeasible solutions within the population at each iteration. This information, represented in Figure 9, is used to put forth the advantage of enabling/disabling the constraint handling during the recombination step of PC1 and PC2.

### 5.4. Comparative Analysis

In the following sections, the results obtained from each scenario and planner configurations, characterized using the types of graphical representation explained in Section 5.3, are analyzed.

#### 5.4.1. Analysis for Scenario 1 (SC1)

For SC1, all the configurations of the planner (PC1-PC3) are tested and their GA are set to run for 100 iterations to illustrate better the convergence behavior of the planner.

Figure 7 summarizes, at three selected iteration counts (in particular at the 1st, 40th, 100th iteration), the evolution of the OF of the population and of the best Pareto front, obtained using PC1. The graphics show: (1) how the number of solutions belonging to the best Pareto front in the population increases as the iteration number grows and (2) how all the solutions move towards the Pareto optimal front, along the axis in the direction of increasing values of the three objective functions. This is the expected behavior of NSGA-II, which is the optimizer that supports the search of the sequence of high-level tasks in our planner. To avoid increasing unnecessarily the length of the paper, it does not include more graphics of this type for the other configurations (PC2 and PC3) or for the remaining scenarios (SC2 and SC3), as they present similar behaviors. Besides, the evolution graphs, used in the rest of the paper are more suitable to provide further insights on the behavior of the planner configurations.

**Figure 8:**
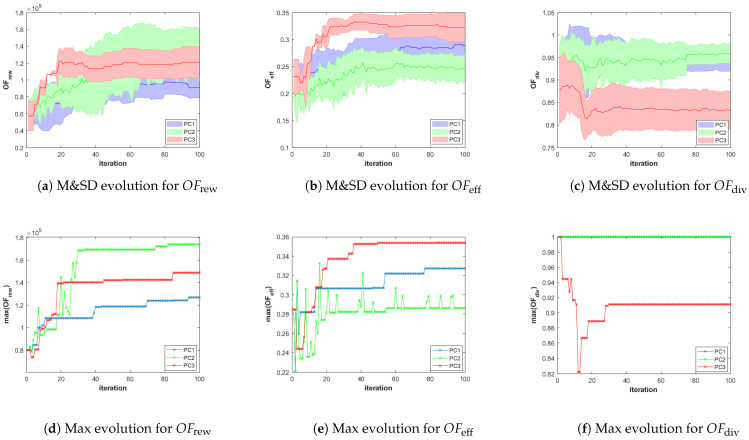
Evolution graphics of the OFs of the feasible solutions of the best Pareto front for SC1. The top row of graphics shows the evolution of the mean and standard deviation (M&SD) of each OF, while the bottom row shows the evolution of the best (Max) value of each OF.

Figure 8a–c show the evolution of the mean and standard deviation of the values of the objective functions ($OF_{\mathrm{rew}}$, $OF_{\mathrm{eff}}$ and $OF_{\mathrm{div}}$) of the feasible solutions that belong to the best Pareto front and that are found using the three planner configurations, while Figure 8d–f show the maximum value of each OF. According to Figure 8a–c, the standard deviations on the values of $OF_{\mathrm{rew}}$, $OF_{\mathrm{eff}}$ and $OF_{\mathrm{div}}$ obtained using PC2 are substantially wider than those obtained with PC1 and PC3. This is due to the fact that the constraint criteria are used only for the parents selection, allowing the MOEA to have a bigger “exploring” capability. Besides, we can also observe that the GA search converges earlier, around iteration 60. Additionally, to show the importance of considering $OF_{\mathrm{div}}$, in SC1 we also test PC3, where $OF_{\mathrm{div}}$ is neglected deliberately during the parents selection and recombination steps. Figure 8c,f show how the diversity criterion evolution is worst for the configuration where $OF_{\mathrm{div}}$ is neglected (that is, for PC3), while PC1 and PC2 reach similar values (in particular the maximum value of $OF_{\mathrm{div}}$ for PC1 is not observed as it is equal to the maximum value of $OF_{\mathrm{div}}$ for PC2). This implies that the mission plans obtained with PC3 suffer from having a low diversity, resulting in a more challenging selection process to be performed by the human operator who is responsible of choosing a “well-balanced” plan among the feasible plans of the best Pareto front returned as plan suggestions by the planner. Besides, if we compare PC1 and PC2, we can conclude that for SC1, PC2 produces overall better solutions regarding $OF_{\mathrm{rew}}$, while PC1 produces overall better solutions with respect to $OF_{\mathrm{eff}}$. However, the values of $OF_{\mathrm{eff}}$ of the solutions found using PC1 is only marginally better than the values of $OF_{\mathrm{eff}}$ of the solutions obtained by PC2, while the values of $OF_{\mathrm{rew}}$ of the solutions obtained with PC2 is significantly bigger than the values of of $OF_{\mathrm{rew}}$ of the solutions obtained with PC1. Therefore, we conclude that PC2, with its better exploring capability, is more suitable for the first scenario.

**Figure 9:**
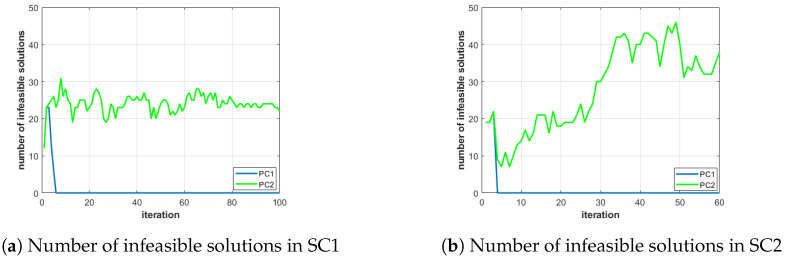
Infeasible plans in the population for PC1 (blue) and PC2 (green) for SC1 (**a**) and SC2 (**b**).

Figure 9a shows that the number of infeasible individuals in the population is also higher in PC2 than in PC1, which practically excludes all infeasible individuals after eight iterations. This demonstrates the difficulty of PC1 to explore new regions of the space that can be reached with the help of some infeasible solutions obtained after random crossover or mutation operations.

**Figure 10:**
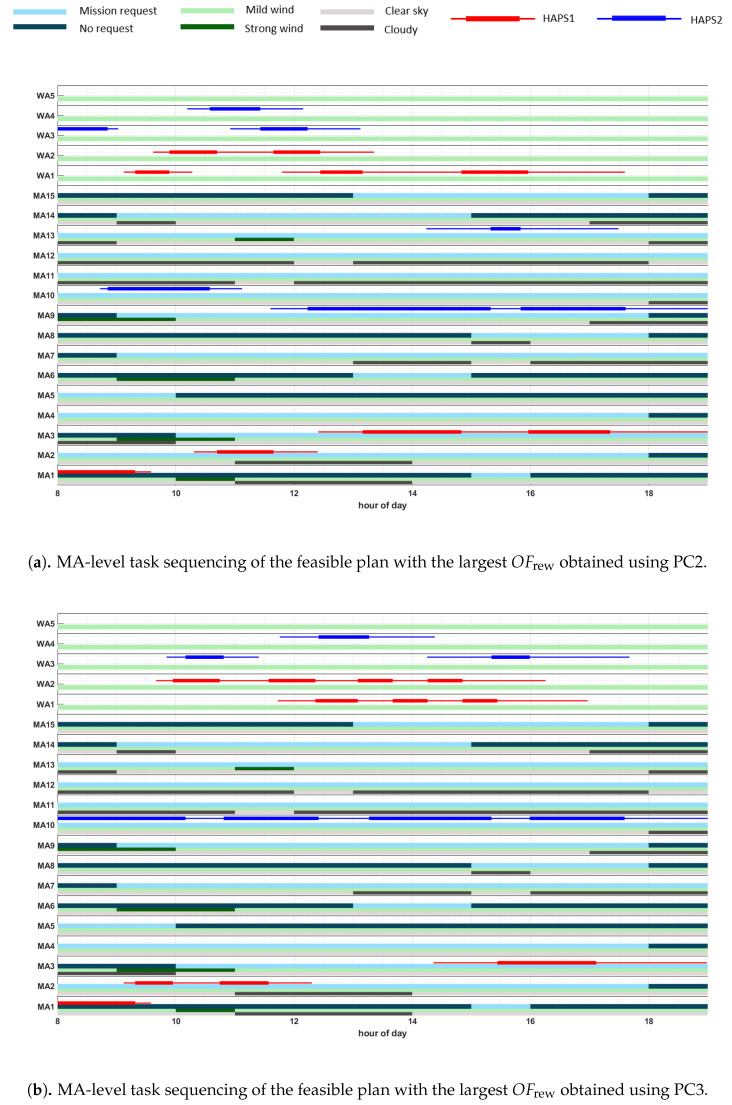
Illustrative examples of feasible plans obtained by PC2 and PC3 for the first scenario (SC1).

Figure 10 shows two representative plans of SC1, which have been selected among the feasible plans of the best Pareto front found using PC2 (in the upper figure) and PC3 (in the lower figure). In particular, we have decided to display the plans that have the maximum $OF_{\mathrm{rew}}$. Comparing the plans of both figures, we can observe that the plan found with PC2 (displayed in Figure 10a) has fewer repetitions of the visited MA than the plan found using PC3 (represented in Figure 10b), where HAPS2 stays monitoring only MA10. This happens because $OF_{\mathrm{div}}$ is neglected in PC3 during the search of the solutions. Additionally, both graphics show how both HAPSs try to accommodate their visit to the MAs to the requested time windows and clear sky weather conditions in order to increment the overall obtained reward. Lastly, by analyzing Figure 8d we can observe that the plan returned by the planner with maximum $OF_{\mathrm{rew}}$ in PC2 has a higher value of $OF_{\mathrm{rew}}$ than the one obtained with PC3, because by including the diversity objective function and by ignoring the constraints in the recombination step, the GA configuration used in PC2 is able to explore the search space more efficiently, jumping to search regions that contain solutions of higher rewards.

Finally, it is worth noting that for the remaining scenarios we do not test against PC3, in order to focus the analysis on the comparison of PC1 and PC2, i.e., the variants of planner configurations that use and ignore the constraint criteria during the recombination of the old and new populations.

#### 5.4.2. Analysis for Scenario 2 (SC2)

In the second scenario, weather data of the same format as the real weather data in SC1 are used but with synthetically increased strong wind. We also set the maximum iterations to 60, which was the iteration number in which the GA converges for SC1.

**Figure 11:**
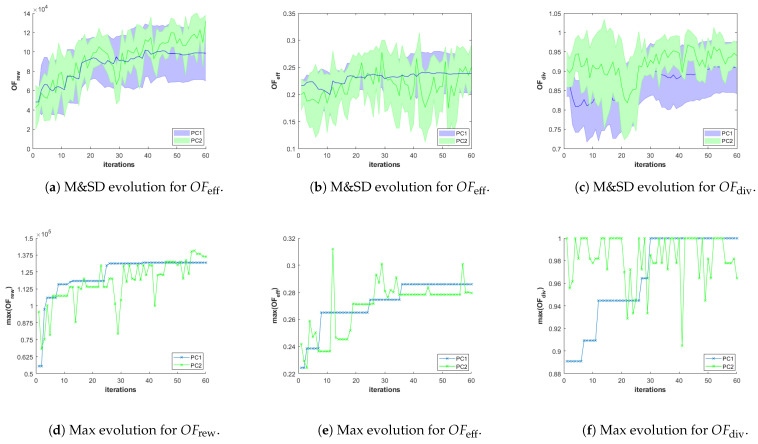
Evolution graphics of the OFs of the feasible solutions of the best Pareto front for SC2. The top row of graphics shows the evolution of the mean and standard deviation (M&SD) of each OF, while the bottom row shows the evolution of the best (Max) value of each OF.

The M&S and Max evolution graphs of the values of the OFs of the feasible solutions belonging to the first Pareto front are shown in Figure 11. The graphics show that the results obtained with respect to the evolution of the objective functions over iterations are comparable in terms of order of magnitude using PC1 and PC2. However, the results obtained with PC2 fluctuate much more than PC1. In fact, the behavior of PC2 is predictable since in this configuration the best Pareto front can obtain both feasible and infeasible solutions, and the feasible solutions, which are the only ones considered for plotting the M&S and Max evolution graphs, can be overtaken by infeasible solutions whose objective function values dominate the objective function values of the feasible solutions.

Although PC2 works well under nominal weather conditions, this planner configuration can be “unstable” under challenging weather conditions, which can facilitate a more frequent violation of the constraints criteria. This behavior can be better explained using Figure 9, where the number of infeasible solutions of the population obtained with PC1 and PC2 for SC1 and SC2 are displayed side-by-side. As already described in the previous subsection, the graphics show how PC1 is much “stricter” against infeasible solutions, as the constraint criteria are used in the recombination step, resulting in a reduction of the “survivability” of infeasible individuals and of the exploring capability of the planner. However, PC2’s higher exploring capability appears to be too “lenient” with the infeasible solutions for SC2, allowing an excessive number of them predominate the population in the final iterations. This behavior, which appears in the more constrained scenario imposed by the stronger winds of SC2, is prone to end up having too few feasible individuals remaining in the best Pareto front of the last iteration, thereby losing the best feasible ones identified along the iterations of the algorithm. Therefore, under more challenging weather conditions, PC1 should be the preferred configuration.

**Figure 12:**
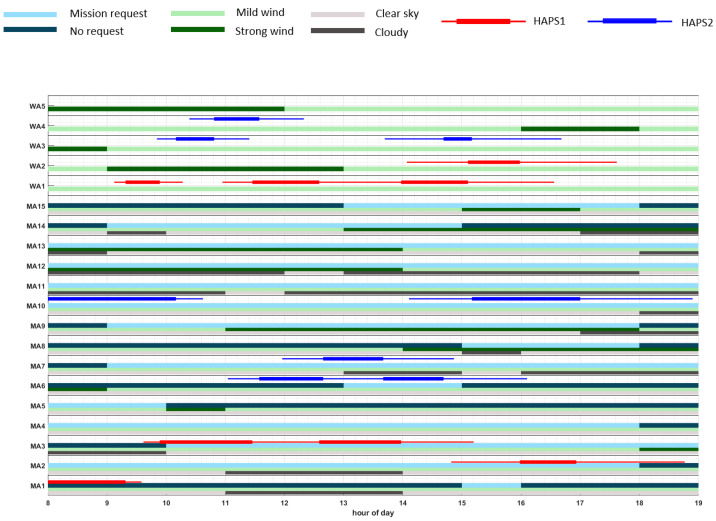
Illustrative example of the feasible MA-level plan with the largest OFrew obtained by PC1 for the second scenario (SC2).

Figure 12 depicts the plan that has the highest expected reward among the feasible plans of the best Pareto front found using PC1 over SC2. The figure shows that the mission elements are affected by strong wind (which occupies more than 20% of the time) more often than in SC1. Besides, HAPS1 monitors MA1 and HAPS2 monitors MA6 at time windows that are not requested by the clients, in order to be able to reach other more promising MAs (and due to the fact that a MA cannot be transversed without monitoring its LOIs).

#### 5.4.3. Analysis for Scenario 3 (SC3)

**Figure 13:**
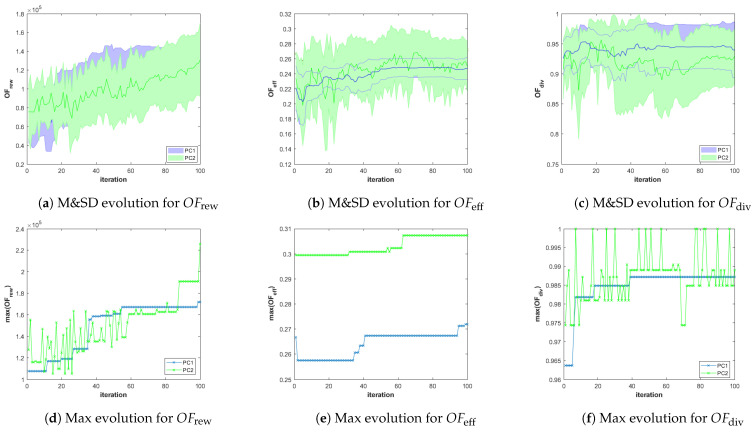
Evolution graphics of the OFs of the feasible solutions of the best Pareto front for SC3. The top row of graphics shows the evolution of the mean and standard deviation (M&SD) of each OF, while the bottom row shows the evolution of the best (Max) value of each OF.

Three HAPSs are used in the third scenario to analyze the scalability of the planner. However, since the search space of the possible solutions has grown (due to the additional HAPS), more iterations of the GA are necessarily. For this reason, we set the maximum iterations of the stop condition to 100, which is also observed to be necessary, as the search takes more iterations to converge according to the evolution graphics of $OF_{\mathrm{rew}}$ presented in Figure 13. Besides, since the nominal weather setting for the environment is used in this scenario (as in SC1), Figure 13 shows how PC2 again exhibits higher variability and better performance than PC1, thanks to its higher “exploring” capability.

**Figure 14:**
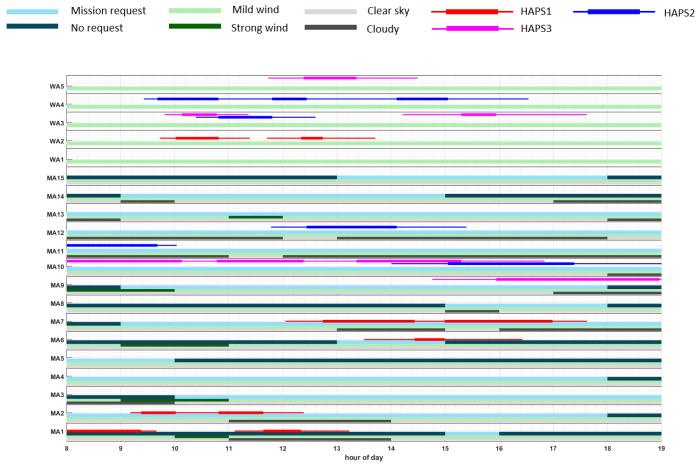
Illustrative example of the feasible MA-level plan with largest $OF_{\mathrm{rew}}$ obtained by PC1 for the third scenario (SC3).

The mission plan with the largest $OF_{\mathrm{rew}}$ among the feasible plans of the first Pareto front found using PC2 is illustrated in Figure 14, along with the operation environment and requirements. With the additional HAPS, more MAs can be monitored, compared to missions where only two HAPS operate (whose illustrative plans are presented in Figure 10 and Figure 12). This fact is also observable comparing the evolution of the Max graphs of $OF_{\mathrm{rew}}$ of SC1 and SC3, because the expected reward obtained by the plans for SC3 (Figure 13d) is higher than the one obtained for SC1 (Figure 8d). Figure 14 also shows how the coexistence in the same MA of multiple HAPS is tolerated (e.g., the presence of HAPS2 and HAPS3 in MA10), since the constraint criterion $\varphi_{\mathrm{coex}}$ is probabilistically evaluated and violated when the probability of coexistence exceeds a given $p_{\mathrm{coex}}$ (which is set to 0.3 in this paper). Changing the value of this parameter, the constraint violation can be “tightened” or “relaxed” as much as desired. This is a novelty of the planner presented in this paper, since the original version presented in [10] implemented a deterministic evaluation of the coexistence criterion where no overlapping of the start and end time range of MA# tasks was allowed.

## 6. Related Works

UAVs have recently become a popular alternative for monitoring ground activities [21], mapping [22] or search and rescue missions [23], since the operation of these platforms is more cost-efficient than using manned aerial vehicles while achieving the same purpose [5]. Furthermore, the deployment of these platforms is also more flexible, because numerous UAV platforms are capable of vertical take-off and landing, enabling the deployment in many missions where vast areas for takeoff and landing are scarce. Lastly, the use of unmanned platforms also allows the immediate deployment in risk zones, without compromising the safety of human pilots nor delaying the operations.

Following the development of battery technologies, light-weight but robust material as well as technologies for optimal harvesting of solar energy, the development of unmanned High Altitude Long Endurance (HALE) aerial vehicles has became the focus of giant aeronautics industries [2,24]. Moreover, new path and mission planning strategies for solar powered UAVs are being continuously developed, intended to (1) improve their trajectory by deriving the most energy-efficient flight patterns [25,26,27,28,29], to (2) determine the optimal path for improving operational efficiency in missions meant for communications [30,31,32], or (3) to track different types of targets [33]. The planning strategies exploited in these works are based on different types of optimization approaches, ranging from nonlinear optimization strategies [26,28,29,30,31] to rapidly-exploring random trees [32], the grasshopper optimization algorithm [33] and particle swarm optimization [27]. Nevertheless, the planners presented in these works either (1) optimize the trajectories without considering any aspect that is relevant to the mission or (2) tackle missions which are significantly different from the one proposed in this paper (and hence they consider a different set of requirements and constraints). Furthermore, the planning methods proposed are different than the GA-based one presented in this paper, although the last two (i.e., [27,33]) are also variants of evolutionary algorithms.

Moreover, the HAPSs considered in this work are special types of HALE platforms aimed to be an alternative to satellites for long-term remote sensing while offering more flexibility in its deployment. With the success stories around Kelleher [2], HAPSs are deemed fit for deployment in the near future at larger scale. However, although HAPSs operations can be beneficial, they can be extremely challenging, given the fragility of the platform under critical weather conditions, their lack of maneuverability, and the requirement for plans for long operations, which oblige the consideration of weather parameters that vary over time within the plan horizon [34]. Specific studies on automated planning for HAPS include [10,35], both aiming to reduce the operators’ workload. In particular, for a complex mission scenario as the one depicted in Figure 1, Ref. [35] proposes a sequential task and motion planning framework for a collective operation area, simplifying the constraints of the planning problem, while [10] uses a GA to extend the temporal hierarchical task planner for multiple HAPSs. Moreover, the current work extends the planner presented in [10] by (1) including the evaluation of the safety and coexistence constraints with the new probability based functions presented in Section 3.3.1 and Section 3.3.2, by (2) substituting the weighted evaluation function used in [10] for the constrained multiobjective Pareto-front evaluation mechanisms of NSGA-II, and by (3) returning the set of the hierarchical plans that form part of the final best Pareto front. Besides, this paper analyzes the behavior of the new planner with new scenario and the influence of the diversity objective function and of different constraint handling techniques within our planner.

Evolutionary algorithms, including variants for solving multiple-objective problems with powerful constraint-handling techniques (such as [19,36]), have often been used for the mission planning of Satellite and UAV operations. For instance, Refs. [37,38,39] present different GA-based planner for scheduling the observation tasks of different satellites, while [23,40,41,42,43] use multiple-objective evolutionary algorithms to solve task planning problems for multiple UAVs engaged in performing monitoring tasks in dissected areas of interest. Although our planner also uses a GA algorithm to determine the best solution plans for a given scenario, it solves a different type of monitoring task mission problem, involving exogenous time-varying events (i.e., weather) and time-dependent mission requirements. Therefore, its evolutionary encoding has a different interpretation and is customized for HAPSs instead of satellites or other types of UAVs. Besides, similar to other works that take into account the uncertainty associated to weather conditions [44,45,46] or to other elements of the mission (e.g., the target location and movement in search and rescue missions [47,48] or the probability of target detection and destruction in hostile environments [49,50]), in this work the uncertainties are incorporated into the models used to evaluate how probable is that each HAPS is at a mission area at a given time, which affects the outcome of the objective and constraint values.

Finally, it is worth highlighting that although this work uses NSGA-II for a constrained multiobjective optimization, it is only a part of the temporal hierarchical task planner, in which the search for optimal decomposition into an ordered list of nonprimitive tasks poses a combinatorial search problem. With an appropriate encoding of the problem at the task level at which the combinatorial problem prevails, NSGA-II is used for guiding the decomposition into executable tasks within a temporal hierarchical task network with a nested Time-Dependent Multi-Vehicle Routing Problem (TDMVRP). Note also that Hierarchical Task Planning often refers to an Artificial Intelligence (AI) planning paradigm and that although there are some domain-independent frameworks meant for it [51,52]; they do not yet support a nested TDMVRP.

## 7. Conclusions and Future Work

This paper presents a new approach for planning the tasks that a group of HAPSs must perform to carry out ground monitoring mission in a structured airspace. The new approach returns a Pareto front of feasible hierarchical plans, whose sequence of higher level tasks is determined using a MOEA that optimizes the expected reward to be received by the HAPSs team for monitoring the different LOIs, the diversity of the LOIs visited by the HAPSs and the time that the HAPSs are actually monitoring (and not traversing the airspace). Besides, it also considers multiple constraints, some encoded in the decomposition method of the hierarchical planner, while others validated by measuring the constraint criteria related to the mission safety, the coexistence of HAPSs in the same MA and the connectivity of the plan. The planner also considers, through the evaluation functions and constraint criteria, the uncertainty that the weather conditions impose on the duration of each task (due to the wind vectors) and on the visibility for the mission camera (due to cloud coverage).

The performance of the different configurations of the planner, carefully set up for increasing/decreasing the “survivability” of the infeasible solutions or to disable the diversity requirement, is tested against several scenarios, with varying number of HAPSs and different weather conditions. The quality of the results is scenario-dependent, although it seems advisable to use the second configuration (PC2) for the planner when the HAPSs operate under mild weather conditions and the first configuration (PC1) for challenging weather conditions, as suggested by the results of the performance tests presented in Section 5. Besides, when planning for two HAPSs the number of iterations required by the planner to converge is smaller than when planning for three HAPSs.

In order to further improve the planner, we will consider several possibilities. Firstly, a “softer” constraint-handling method can be used to improve PC1. For this purpose, we are planning to adopt the approach proposed by [20], in which, with a low probability, some infeasible solutions can be ranked better than feasible solutions. This new planner configuration could avoid, for example, the early convergence of PC1 in SC2, while still managing to maintain the right balance between feasible and infeasible solution plans in the population.

Secondly, while the planner is typically customized for solving HAPS mission planning problems, it can be extended for more generic uses. As a matter of fact, temporal hierarchical task planners (without a nested TDMVRP) are gearing toward general implementation [52]. Hence, with careful considerations of the encoding of the chromosomes for generic planning problems and more generic approaches for tuning the planner parameters, the approach presented in this work could be implemented in a “domain-independent” fashion.

Finally, although the underlying mission planner reduces the operator’s workload, when the planner suggest many solutions, selecting the one to execute can still be challenging for the operator. Therefore, in order to increase usability of the planner, it can be convenient to provide operators with a set of tools that help them to analyze the solutions of the best Pareto front more easily, by (1) taking into account the explicability of the plans with a visualization interface that can highlight the probable constraint violations, the rewarding mission tasks, the diversity of the clientele pool and the effort; or by (2) designing filter mechanisms that accelerate the selection of the plan of the best Pareto front that better fits the operator preference (e.g., the one with the maximum value of an objective function or the one with the best preference weighting [53]). In a similar line, more interactive functions can be integrated to enable “mixed-initiative planning”, which can favor quick local replanning performed by the operator whenever necessary, due to unexpected weather change or to take into account the operator’s preferences.

## Figures and Tables

**Table 1:** Relationships among characteristics of HAPSs and their benefits (+) and challenges (−).

Properties	Benefits and Challenges during Operation
Light-weight material	(+) Energy efficient (−) Fragile and vulnerable to adverse weather
Limited payload	(+) Energy efficient (−) Limited onboard computation power
Fixed-wing, large wingspan	(+) More surface for harvesting solar power (−) Limited maneuverability with respect to turn rate and mid-air still-stop
High flight altitude	(+) Calmer weather (−) Takeoff and landing are time consuming
Extreme long endurance	(+) Suitable for longer missions (+) No frequent takeoff and landing necessary (−) High operating cost
Low-power electro-motor, low airspeed	(+) Energy efficient (−) Wind effect cannot be neglected

**Table 2:** Scenarios considered for performance tests.

Scenarios	Weather Data	Number of HAPSs
SC1 (Nominal scenario)	Historical (April 2018)	2
SC2 (Challenging weather scenario)	With synthetically increased wind magnitude	2
SC3 (Three HAPSs scenario)	Historical (April 2018)	3

**Table 3:** Planner configuration parameters.

Planner Parameters	Parameter Values
Crossover probability, $p_{\mathrm{crossover}}$	0.9
Mutation probability, $p_{\mathrm{mut}}$	0.1
Population size, $N_{P}$	50
Tournament size, $k_{\mathrm{tournament}}$	3
Number of generations, $I_{\max}$	100 (in SC1 and SC3), 60 (in SC2)
**Constraint Thresholds**	**Threshold Values**
$p_{\mathrm{saf}}$	0.1
$p_{\mathrm{coex}}$	0.3

## Data Availability

Not applicable.

## Abbreviations

The following abbreviations are used in this manuscript:

AI	Artificial Intelligence
C	Corridor
EA	Evolutionary Algorithm
EO	Electro-Optical
FL	Flight Level
GA	Genetic Algorithm
GCS	Ground Control Station
HALE	High Altitude Long Endurance
HAPS	High Altitude Pseudo-Satellite
HFR	High-level Flight Rules
LOI	Location Of Interest
MA	Mission Area
MC	Mission Constraint
MOEA	Multi-Objective Evolutionary Algorithm
MR	Mission Requirement
NSGA	Nondominated Sorting Genetic Algorithm
PC	Planner Configuration
TDMVRP	Time Dependent Multi-Vehicle Routing Problem
UAV	Unmanned Aerial Vehicle
WA	Waiting Area
WP	Waypoint

## Appendix A. Numerical Details on the HAPS Model and on the Mission Parameters

Table A1 details numerical information on the HAPS that are assumed in this work.

**Table A1:** Model of the HAPS considered in this work: build, performance, and mission payload.

Build/Fight Performance/Payload	Parameter Values
Weight	100 kg
Wingspan	30 m
Payload	5–10 kg
Battery capacity	15 kWh
Electro-motor maximum propulsive power	1700 W
Operating altitude	18 km
Cruise airspeed at the operating altitude	30 m/s
Endurance	3 months
Ground sampling distance at 18 km	30 cm
hxw of an image	360 × 3000 m

The dimensions of the mission elements depicted in Figure 1 in form of their longest diagonals in kilometers are given in Table A2.

**Table A2:** Dimensions of the mission elements depicted in Figure 1.

C	Longest Diagonal [km]	MA/WA	Longest Diagonal [km]	LoI 1	LoI 2	LoI 3
**C1**	60.78	**MA1**	27.58	17.10		
**C2**	37.83	**MA2**	69.49	19.83		
**C3**	33.67	**MA3**	103.11	20.07	44.27	
**C4**	58.88	**MA4**	25.32	15.56		
**C5**	34.22	**MA5**	52.90	13.25		
**C6**	17.64	**MA6**	46.51	21.61		
**C7**	70.41	**MA7**	84.03	21.42	25.88
**C8**	40.27	**MA8**	36.62	10.84		
**C9**	66.19	**MA9**	133.30	34.36	21.24	38.13
**C10**	72.10	**MA10**	123.35	39.96	44.74	
**C11**	20.52	**MA11**	74.98	31.62	24.53	
**C12**	73.35	**MA12**	73.49	18.28	26.38	
**C13**	39.34	**MA13**	56.87	18.62		
**C14**	88.46	**MA14**	36.28	14.31		
**C15**	63.04	**MA15**	104.92	23.66	36.46	
**C16**	70.70	**WA1**	47.90			
**C17**	71.98	**WA2**	34.44			
**C18**	71.38	**WA3**	46.41			
**C19**	61.77	**WA4**	47.96			
**C20**	42.75	**WA5**	26.44			
**C21**	46.67					
**C22**	39.80					
**C23**	74.26					
**C24**	38.15					
**C25**	106.86					
**C26**	63.86					

The rewards obtained for the successful monitoring of all LOI(s) of a MA are listed in Table A3, along with the image coverage of the ground required for the monitoring mission to be considered successful.

**Table A3:** Rewards to be given for each MA (× $10^{3}$).

Mission Area	Coverage (%)	Reward (€)	Mission Area	Coverage (%)	Reward (€)
**MA1**	80	4	**MA9**	60	13
**MA2**	80	50	**MA10**	60	18
**MA3**	60	100	**MA11**	70	20
**MA4**	80	20	**MA12**	70	10
**MA5**	80	3	**MA13**	60	8
**MA6**	70	5	**MA14**	90	18
**MA7**	70	15	**MA15**	50	9
**MA8**	80	3			
